# Blind spots of universal primers and specific FISH probes for functional microbe and community characterization in EBPR systems

**DOI:** 10.1093/ismeco/ycae011

**Published:** 2024-01-23

**Authors:** Jing Yuan, Xuhan Deng, Xiaojing Xie, Liping Chen, Chaohai Wei, Chunhua Feng, Guanglei Qiu

**Affiliations:** School of Environment and Energy, South China University of Technology, 382 Waihuandong Road, University Town, Guangzhou, Guangdong 510006, China; School of Environment and Energy, South China University of Technology, 382 Waihuandong Road, University Town, Guangzhou, Guangdong 510006, China; School of Environment and Energy, South China University of Technology, 382 Waihuandong Road, University Town, Guangzhou, Guangdong 510006, China; School of Environment and Energy, South China University of Technology, 382 Waihuandong Road, University Town, Guangzhou, Guangdong 510006, China; School of Environment and Energy, South China University of Technology, 382 Waihuandong Road, University Town, Guangzhou, Guangdong 510006, China; Guangdong Provincial Key Laboratory of Solid Wastes Pollution Control and Recycling, 382 Waihuandong Road, University Town, Guangzhou, Guangdong 510006, China; Key Laboratory of Pollution Control and Ecological Restoration in Industrial Clusters, Ministry of Education, 382 Waihuandong Road, University Town, Guangzhou, Guangdong 510006, China; School of Environment and Energy, South China University of Technology, 382 Waihuandong Road, University Town, Guangzhou, Guangdong 510006, China; Guangdong Provincial Key Laboratory of Solid Wastes Pollution Control and Recycling, 382 Waihuandong Road, University Town, Guangzhou, Guangdong 510006, China; Key Laboratory of Pollution Control and Ecological Restoration in Industrial Clusters, Ministry of Education, 382 Waihuandong Road, University Town, Guangzhou, Guangdong 510006, China; School of Environment and Energy, South China University of Technology, 382 Waihuandong Road, University Town, Guangzhou, Guangdong 510006, China; Guangdong Provincial Key Laboratory of Solid Wastes Pollution Control and Recycling, 382 Waihuandong Road, University Town, Guangzhou, Guangdong 510006, China; Key Laboratory of Pollution Control and Ecological Restoration in Industrial Clusters, Ministry of Education, 382 Waihuandong Road, University Town, Guangzhou, Guangdong 510006, China

**Keywords:** biological enhanced phosphorus removal (EBPR), fluorescence in situ hybridization (FISH), 16S rRNA gene amplicon sequencing, polymerase chain reaction, primers, probes, polyphosphate accumulating organisms (PAOs), glycogen accumulating organisms (GAOs)

## Abstract

Fluorescence in situ hybridization (FISH) and 16S rRNA gene amplicon sequencing are commonly used for microbial ecological analyses in biological enhanced phosphorus removal (EBPR) systems, the successful application of which was governed by the oligonucleotides used. We performed a systemic evaluation of commonly used probes/primers for known polyphosphate-accumulating organisms (PAOs) and glycogen-accumulating organisms (GAOs). Most FISH probes showed blind spots and covered nontarget bacterial groups. *Ca*. Competibacter probes showed promising coverage and specificity. Those for *Ca*. Accumulibacter are desirable in coverage but targeted out-group bacteria, including *Ca*. Competibacter, *Thauera*, *Dechlorosoma*, and some polyphosphate-accumulating Cyanobacteria. *Defluviicoccus* probes are good in specificity but poor in coverage. Probes targeting *Tetrasphaera* or *Dechloromonas* showed low coverage and specificity. Specifically, DEMEF455, Bet135, and Dech453 for *Dechloromonas* covered *Ca*. Accumulibacter. Special attentions are needed when using these probes to resolve the PAO/GAO phenotype of *Dechloromonas*. Most species-specific probes for *Ca*. Accumulibacter, *Ca*. Lutibacillus, *Ca*. Phosphoribacter, and *Tetrasphaera* are highly specific. Overall, 1.4% *Ca*. Accumulibacter, 9.6% *Ca*. Competibacter, 43.3% *Defluviicoccus*, and 54.0% *Dechloromonas* in the MiDAS database were not covered by existing FISH probes. Different 16S rRNA amplicon primer sets showed distinct coverage of known PAOs and GAOs. None of them covered all members. Overall, 520F-802R and 515F-926R showed the most balanced coverage. All primers showed extremely low coverage of *Microlunatus* (<36.0%), implying their probably overlooked roles in EBPR systems. A clear understanding of the strength and weaknesses of each probe and primer set is a premise for rational evaluation and interpretation of obtained community results.

## Introduction

Enhanced biological phosphorus removal (EBPR) is an efficient and sustainable process widely used for phosphorus (P) removal in municipal wastewater treatment plants (WWTPs) [1-6]. The process relies on the occurrence and enrichment of microorganisms capable of excessively storing polyphosphate (poly-P) (i.e. the polyphosphate-accumulating organisms, PAOs) [7-10]. In the anaerobic stage, PAOs obtain energy by consuming intracellular poly-P and glycogen, taking up carbon sources (such as volatile fatty acids, VFAs), and storing them in the form of intercellular storage compounds (such as polyhydroxyalkanoates, PHA). In the aerobic stage, PAOs grow on the anaerobically stored carbon sources, replenish glycogen and take up orthophosphate, achieving P removal [11-13].

Apart from PAOs, microorganisms of interest in the EBPR process include glycogen-accumulating organisms (GAOs), the metabolic characteristics of which are similar to PAOs, except that they store glycogen but not poly-P [14-16]. Most known PAOs and GAOs remain un-culturable, highlighting the necessity to use biomolecular techniques to study their occurrence and relative abundances in complex communities [17]. Additionally, the functional traits of these microorganisms are typically conserved at low taxonomic levels (genus, species, or strain). Precise *in-situ* characterization is extremely essential to understand their occurrence and dynamics, and to reveal their metabolic characteristics [18-20].

**Figure 1:**
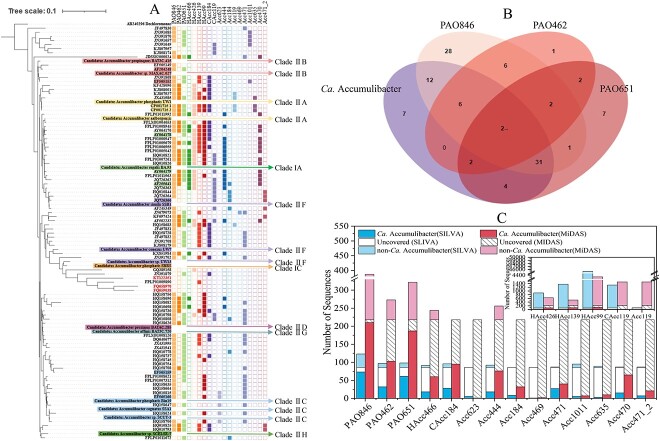
Coverage of commonly used FISH probes for *Ca*. Accumulibacter. A. A phylogenetic tree of ca. Accumulibacter showing the coverage of each probe. The tree was built using refined reference sequences obtained from the SILVA database (Figure S1). Not all the sequences were originally recovered from activated sludge. Color parts of the outer circle indicate the sequences that were covered by each probe. The sequences which were not covered by exsiting probes were marked in different font colors (detailed origination information of these sequences is given in Table S2). 16S rRNA gene sequences from metagenome-assembled genomes (MAGs) were inserted into the tree (denoted with different fill colors) to show the possible clade affilination of each sequence [60, 78]. The maximum likelihood method was used with the Tamura-Nei model and none branch swap fitter for tree construction. The scale bar represents substitutions per nucleotide base. B. A Venn diagram showing the covered and/or co-covered number of *Ca*. Accumulibacter sequences (with refined reference sequences obtained from the SILVA database) by each probe. C. The numbers of target and nontergeted sequences which were covered by each probe in the SILVA and the MiDAS databases.

Fluorescence in situ hybridization (FISH) and 16S rRNA gene amplicon sequencing based techniques are commonly used for *in-situ*, specific, and/or high-level taxonomic resolution analyses of functional bacteria in EBPR systems. Being introduced in the 1980s [21, 22], FISH is based on oligodeoxynucleotide probes complementary to ribosomal RNA sequences of a specific lineage of microorganisms. PAOmix (a mixture of PAO462, PAO651, and PAO846, Fig. 1) was widely used to target *Candidatus* Accumulibacter-related PAOs [18, 20]. Actino-1011 was first designed to target *Tetrasphaera*-related PAOs [23], followed by Actino-658 and Actino-221 for different sub-groups [24]. Apart from the Actino-221 and Actino-658 defined ones, *Tetrasphaera* with different morphologies were observed in full-scale WWTPs. Probes targeting different phylogenetic clusters were thus designed (i.e. Elo1–1250, Tet1–823, Tet1–266, Tet2–842, Tet2–831, Tet2–892, Tet2–87, Tet2–174, Tet3–654, and Tet3–19), endowing the characterization and identification of the ecological niches of distinct lineage members [25]. Recently, Phos601, Phos741, and Luti617 were developed to target and define novel *Tetrasphaera*-related PAOs (i.e. *Ca*. Phosphoribacter and *Ca*. Lutibacillus) which were previously recognized as Clade 3 *Tetrasphaera* [26]. The previously designed probe Actino-658 was found to cover mainly *Ca*. Phosphoribacter. *Ca*. Phosphoribacter and *Ca*. Lutibacillus were shown to be more abundant than *Tetrasphaera* in EBPR systems globally [26]. GAO431 and GAO989 were designed to target γ-Proteobacteria related GAOs. The targeted group was named *Ca*. Competibacter phosphatis [14]. These two probes are widely used for *in-situ* identification and characterization of *Ca*. Competibacter. Recently, for improved coverage and specificity in the FISH-related identification of *Ca*. Competibacter GAOs, CPB-654 was designed [27]. Other commonly used probes include DF1004, DF1013, DF198, DF1020, DF988, TFO_DF862, TFO_DF618, TFO_DF218, and DF181 for *Defluviicoccus* [28-31]. Dech443, DCMAG455, and DEMFE455 for *Dechloromonas* [32, 33]. Bet135 for undescribed Rhodocyclaceae members [34]. To understand the eco-physiological characteristics of these PAOs and GAOs, FISH may be combined with chemical staining, microautoradiography, Raman microscopy, and other *in-situ* techniques. The carbon source utilization, P release and uptake, and intercellular storage compound dynamics under designated conditions may be resolved *in-situ* [10, 26, 31, 35-37].

For FISH and associated analysis, the design of oligonucleosides (probes) that could unbiasedly target a designated group of microorganisms is essential but challenging. There is a trade-off between coverage and specificity [38]. Few probes could perfectly cover a specific group of bacteria without targeting outgroup microorganisms. A detailed and correct understanding of the coverage and specificity of a specific probe is a prerequisite for rational interpretation of the obtained results. Whereas, there is a lack of a comprehensive and systematic understanding of the pros and cons of commonly used FISH probes for functional microorganisms (PAOs and GAOs) in the EBPR systems.

Apart from FISH, 16S rRNA gene-based methods (including the commonly used 16S rRNA gene amplicon sequencing) are also widely used for community analysis in EBPR systems. Most 16S rRNA gene-based methods are based on polymerase chain reaction (PCR) amplification of the full-length or fragment of the 16S rRNA gene using universal primers. Primer selection is a key factor affecting PCR amplification and the rational reflection of the microbial community structures [20, 39]. Using non-optimized primer sets may result in the miss-out of certain species or genera [40, 41]. For community composition research, it is ideal to have primers that could non-selectively target all microorganisms. However, no existing primers could indiscriminately amplify a target DNA section from all community members [40]. The effects of 16S rRNA gene primer sets and their respective covered regions on bacterial community diversity were analyzed for activated sludge bacterial community characterization [42]. Results showed that V3-V4 regions targeted primer set (338F-802R) seemed to have the highest coverage with low bias at the genus level. However, Albertsen *et al*. [39] suggested that V1-V3 seemed to be an ideal region for the analysis of the activated sludge community. Despite these studies, it is still unknown which set of primers performs the best for EBPR related communities. For EBPR, effective, complete, and indiscriminate capture of functional groups was more meaningful than enumerating the entire bacterial community. Otherwise, the analysis may result in commonly occurring blind spots even in well-known functional groups. It is necessary to well-understand the coverage of universal primers on known PAOs and GAOs.

In this study, we systematically evaluated the coverage and specificity of commonly used FISH probes for known PAOs and GAOs together with a systematic evaluation of coverage of commonly used 16S rRNA gene amplicon primers, to identify the blind spots of these commonly used techniques in capturing and analyzing each taxon of PAOs and GAOs. The results are expected to provide base points for pertinent understanding and interpretation of the 16S rRNA amplicon sequencing and FISH results in EBPR research. A quantitative and detailed elaboration of the dead zones of each technique would also benefit in uncovering the potentially hidden communities in each functional taxon in EBPR systems.

## Material and methods

### Data sources in the SILVA and MiDAS databases

16S rRNA gene sequences, including those from *Ca*. Accumulibacter, *Tetrasphaera*, *Dechloromonas*, *Microlunatuas*, *Ca*. Competibacter, *Defluviicoccus*, etc. were retrieved from the SILVA 16S rRNA gene non-redundant reference (SSU r138.1 RefNR) database (https://www.arb-silva.de/download), and the MiDAS 4.0 database (https://www.midasfieldguide.org). These bacteria are known as important PAOs and/or GAOs in EBPR systems and thus were focused in this study for the analyses of the coverage and/or specificity of 16S rRNA gene amplicon primers and FISH probes.

FISH probes (Table 1) evaluated in this study were obtained from the probeBase database (http://probebase.csb.univie.ac.at/) and literatures. For the probeBase database, specific probes and sequences were searched by entering the target bacteria name (https://probebase.csb.univie.ac.at/pb_search). The probe coverage and the covered sequences were evaluated and obtained via SILVA TestProbe (https://www.arb-silva.de/search/testprobe/) with parameters: SSU r138.1 (SILVA database), RefNR (Sequence Collection), 0 mismatches (Maximum Number of Mismatches), and 0 N’s (Consider × occurrences of N (aNy nucleotide) as match).

**Table 1:** Commonly using FISH probes targeting different groups of PAOs and GAOs in EBPR systems.

	Probe name	Sequence (5′-3′)	SILVA SSU r138.1 refNR			MiDAS			Target group	FA(%)^a^	Reference
			Coverage (%)	Non-target hits	Targeted hits	Coverage (%)	Non-target hits	Targeted hits			
*Ca*. Accumulibacter	Acc444	CCCAAGCAATTTCTTCCC	20.9	6	18	34.9	38	76	Clade IA	35	[67]	
	HAcc466	CATCTACTCAGGGTATTAA	20.9	5	18	27.6	26	60		35		
	HAcc426	CGCCGAAAGAGCTTTACA	30.2	810	26	42.2	418	92		35		
	Acc184	GCTCCCAGAACGCAAGGT	9.3	0	8	14.7	0	32	Clade IF	35		
	CAcc184	GCTCCCAGAGCGCAAGGT	32.6	10	28	43.6	0	95		35		
	Acc119	GGATACGTTCCGATGCTT	5.8	8	5	8.3	1269	18	Clade IIA	35		
	HAcc99	CTCACCCGTCCGCCACTC	38.4	45 262	33	50.9	4097	111		35		
	HAcc139	GCTACGTTATCCCCCACTC	33.7	1279	29	44.5	291	97		35		
	CAcc119	GGGCACGTTCCGATGCAT	19.8	1233	17	8.3	1269	18		35		
	Acc623	CCAGCTGGACAGTCTCAA	5.8	0	5	0.5	0	1	Clade IIC	35	[68]	
	Acc469	CCAGGTACCGTCATCTACACAGGC	1.2	0	1	0.9	0	2	*Ca*. Accumulibacter proximus	30	[20]	
	Acc471	CTCCAGGTACCGTCATCTACACAG	31.4	1	27	18.3	0	40	*Ca*. Accumulibacter affinis	40		
	Acc1011	GCGAGCACTCCCAGATCTCTC	5.8	9	5	3.2	0	7	*Ca*. Accumulibacter propinquus	40		
	Acc635	AACTCCAGCCTGGCAGTCTCAAAT	3.5	3	3	4.6	0	10	*Ca*. Accumulibacter regalis	30		
	Acc470	TTCGGGTACCGTCATCTACTCAGG	17.4	1	15	29.8	0	65	*Ca*. Accumulibacter aalborgensis	30		
	Acc471_2	AGTCGGGTACCGTCATCTACACAG	8.1	1	7	9.6	0	21	*Ca*. Accumulibacter iunctus，*Ca*. Accumulibacter similis	30		
	PAO846	GTTAGCTACGGCACTAAAAGG	84.9	37	73	96.3	169	210	*Ca*. Accumulibacter	35	[18]	
	PAO462	CCGTCATCTACWCAGGGTATTAAC	37.2	11	32	47.2	55	103		35		
	PAO651	CCCTCTGCCAAACTCCAG	70.9	12	61	85.8	104	187		35		
*Tetrasphaera*	Tet1–266	CCCGTCGTCGCCTGTAGC	0	0	0	0	0	0	Clade 1	25	[25]	
	Tet2–174	GCTCCGTCTCGTATCCGG	7.8	6	4	7.6	1	12	Clade 2	20		
	Tet2–831	TCGTGAAATGAGTCCCAC	17.6	0	9	15.3	0	24		10		
	Tet2–842	GCGGCACAGAACTCGTGA	19.6	1	10	23.6	0	37		30		
	Tet2–87	TCGCCACTGATCAGGAGA	2	11	1	4.5	19	7		10		
	Tet2–892	TAGTTAGCCTTGCGGCCG	0	0	0	0	0	0		5		
	Tet3–19	CAGCGTTCGTCCTACACA	0	1	0	0	0	0	Clade 3	0		
	Tet3–654	GGTCTCCCCTACCATACT	2	16	1	0	26	0		35		
	Elo1–1250	CGCGATTTCGCAGCCCTT	19.6	9	10	28	2	44	Clade 1	20		
	Actino-221	CGCAGGTCCATCCCAGAC	2	0	1	3.2	0	5	Actinobacterial PAO	30	[24]	
	Tet2–823	TGAGACCCGCACCTAGTT	0	0	0	0	0	0	Clade 2	30	[25]	
	Actino-1011	TTGCGGGGCACCCATCTCT	39.2	170	20	41.4	65	65	Epbr19, Ebpr20	30	[23]	
	Tetra67^c^	AGCAAGCTCCTCCGTCACCG	5.9	15	3	22.3	11	35	midas_s_299, midas_s_469, *Tetrasphaera elongata*, midas_s_24955, midas_s_24809, midas_s_35051, midas_s_5540, midas_s_31199	40	[26]	
	Tetra732	AGTGGTGGCCCAGAGACCTG	5.9	3	3	9.6	0	15	midas_s_299, midas_s_328, midas_s_1378	40		
	Tetra183	TAGAGATGCCTCTCCGTCTC	35.3	39	18	58.6	72	92	*Tetrasphaera*	30		
*Ca*. Phosphoribacter^b^	Actino-658	TCCGGTCTCCCCTACCAT	60.0	2	15	35.1	0	26	*Ca*. Phosphoribacter	40	[24]	
	Phos741	TTCTCAGCGTCAGTTGTGGCCC	21.4	3	6	51.4	0	38		30	[26]	
	Phos601	GGTTGAGCCTCGGATTTTCACTGC	21.4	0	6	9.5	0	7	*Ca*. Phosphoribacterhodrii	30		
*Ca*. *Lutibacillus*^b^	Luti617	CCCACTGCAAGTCCGGAATTGAGT	60.0	0	3	33.3	0	5	midas_s_45	30	[26]	
*Defluviicoccus*	TFO_DF862	AGCTAAGCTCCCCGACAT	5.3	1	4	11	0	18	*Defluviicoccus vanus*	35	[31]	
	TFO_DF618	GCCTCACTTGTCTAACCG	5.3	0	4	2.4	0	4	Cluster I	25–35		
	TFO_DF218	GAAGCCTTTGCCCCTCAG	9.3	3	7	18.3	0	30		25–35		
	DF1004	TAAGTTTCCTCAAGCCGC	1.3	0	1	2.4	0	4	C17 & C23 clones	35	[30]	
	DF1013	GAACTGAAGGCTCGAGTTTC	1.3	0	1	4.3	0	7	A40 & B29 clones	35		
	DF198	ATCCCAGGGCAACATAGTCT	4	0	3	9.8	0	16	*Ca*. Monilibacter batavus-related organisms	35		
	DF181B	CTTTGCCCCTCAAGGCAC	4	0	3	4.9	0	8	Cluster IV	30	[28]	
	DF181A	CTTTCCCTCACAAGGCAC	1.3	0	1	1.2	0	2	Cluster IV	30		
	DF1020	CCGGCCGAACCGACTCCC	9.3	8	7	23.2	2	38	Cluster II	35	[29]	
	DF988	GATACGACGCCCATGTCAAGGG	10.7	0	8	17.7	0	29		35		
*Dechloromonas*	DCMAG455	CAGGTATTAGCTGATGCG	3.3	6	6	6.5	0	25	*Dechloromonas agitata*	30	[32]	
	DEMFE455	AGGGTATTAACCCATGCG	22.1	48	40	21.4	61	83	*Ferribacterium limneticum*, few *Dechloromonas* spp.	30		
	Bet135	ACGTTATCCCCCACTCAATGG	8.9	35	16	15.5	23	60	Skagen clones 42 and 76, and eight closely related Rhodocyclaceae clones	45	[34]	
	Dech453	GGGTATTCACCCATGCGA	2.8	2	5	0.8	1	3	*Dechloromonas*	35	[68]	
	Dech443	ACCCATGCATTTTCTTCCCGG	3.3	0	6	8	0	31	*Dechloromonas* sub-group	35	[33]	
*Ca*. Competibacter	CPB654	TCCTCTAGCCCACTC	87.4	22	83	90.4	76	388	Competibacter lineage	35	[27]	
	GAO431	TCCCCGCCTAAAGGGCTT	57	6	48	51	0	219	*Ca*. Copmetibacter phosphatis	35	[14]	
	GAO989	TTCCCCGGATGTCAAGGC	59	6	50	50.1	1	215		35–55		

a [FA] = formamide concentration in hybridization buffer.

b Evaluation was performed base on currently identified 16S rRNA gene sequences of *Ca*. Phosphoribacterb *Ca*. Lutibacillusb.

c It is suspected that the sequence of Tetra67 given in the original publication [26] might have missed three bases in between. After adding three bases, the probe generated similar results as reported in the original publication. The sequence given here is the suspected correct one.

Universal primers (Table S1, see online supplementary material for a colour version of this table), targeting different regions of the bacterial 16S rRNA genes, including 27F-533R and 27F-534R for V1-V3 [39, 43], 338F-806R [44] and 341F-806R [45] for V3-V4, 520F-802R [46] and 515F-806R [47] for V4, 515F-907R [48] and 515F-926R [47] for V4-V5, and 799F-1193R [49] for V5-V7, were obtained from literatures as well as from the SILVA database. The coverage of these universal primers on each phylogenetic group of PAOs and GAOs was analysed with a mismatch of 0 and other default settings for each primer set against the SILVA SSU r138.1 RefNR (https://www.arb-silva.de/). Primer coverages and uncovered sequences were obtained. BLASTn was employed to compare all probes and primers against the MiDAS database with the BLASTn-short parameter [50, 51] and a tolerance of 0 mismatch. Scripts were compiled and documented in the Supplementary 2 Scripts (Text S1). For the evaluation of the primer sets containing 27F, 16S rRNA gene sequences that do not have sequences at the 27F end was omitted.

### Phylogenetic analysis

A robust phylogeny analysis and reliable taxonomic assignment of the target bacteria lineage is a prerequisite for the evaluation of the performances of probes. In view of the relatively complicated phylogeny of *Ca*. Accumulibacter and *Propionivibrio* [20], and the recent advances in redefining the Clade 3 *Tetrasphaera* as two novel genera: *Ca*. Phosphoribacter and *Ca*. Lutibacillus [26], phylogenetic analyses (Figure S1, see online supplementary material for a colour version of this figure) were performed on *Ca*. Accumulibacter, *Propionivibrio*, *Ca*. Phosphoribacter, *Ca*. Lutibacillus, and *Tetrasphaera* related sequences which were retrieved from the SILVA and MiDAS databases to confer that the collection of sequences used for probe performance evaluation were indeed affiliated to respective taxa. 16S rRNA gene sequences with confirmed taxonomic assignments (including those of pure isolates, from complete genomes and/or metagenome-assembled genomes (MAGs), and those used in previous researches [20, 26]) were inserted into the phylogenetic trees (Figure S1, see online supplementary material for a colour version of this figure) which were built using the references sequences retrieved from the SILVA SSU r138.1 RefNR and the MiDAS 4.0 databases. Two aspects were comprehensively evaluated to determine and confirm the taxonomy of each reference 16S rRNA gene sequence: (i) 16S rRNA gene sequences with confirmed taxonomic assignments served as references for the 16S rRNA genes obtained from the SILVA and MiDAS databases. (ii) Clustering analysis was performed on each collection of 16S rRNA gene sequences using CD-HIT [52] with a sequence identity cut-off of 94.5%, the sequences clustered in each cluster are carefully evaluated for their taxonomy [53].

After confirming the taxonomic assignment of each reference sequences, CD-HIT [52] was employed to cluster each collection of confirmed 16S rRNA gene sequences with a sequence identity cut-off of 98.7%, which was regarded as a boundary of species [54]. For each species, 16S rRNA gene sequences were aligned by using MAFFT with default parameters [55]. The alignments were inputted into IQ-TREE v2 to construct phylogenetic maximum likelihood trees, with 1000 ultrafast bootstrap approximation. In the following specificity and coverage study of primer sets and probes, the refined *Ca*. Accumulibacter, *Propionivibrio*, *Ca*. Phosphoribacter, *Ca*. Lutibacillus, and *Tetrasphaera* sequences (Figure S1, see online supplementary material for a colour version of this figure), and other untreated PAOs/GAOs (*Dechloromonas*, *Ca*. Competibacter, and *Defluviicoccus*) sequences were used. The origination information of the sequences (with respect to whether or not they were retrieved from activated sludge) which were not covered by existing probes is given in Table S2–S6, see online supplementary material for a colour version of this figure.

For intuitive illustrations of the coverage of each probe, sequences in the SILVA SSU r138.1 RefNR database (including the refined ones for *Ca*. Accumulibacter and *Tetrasphaera*, and untreated ones for other PAOs/GAOs, as mentioned above) were used to build phylogenetic trees (the MiDAS database has a huge collection of non-redundant sequences for each taxon, thus was not used for phylogenetic tree illustration). The phylogenetic trees were constructed via MEGA 7 and iTOL (https://itol.embl.de/) using the maximum likelihood method with the Tamura-Nei model and none branch swap fitter.

### Sampling in lab- and full-scale wastewater treatment systems

Twenty-six activated sludge samples were collected from a lab-scale sequencing batch reactor (SBR) and a full-scale WWTP in Guangzhou, China. The lab-scale SBR with a working volume of 5 L was inoculated with activated sludge collected from the same WWTP. The SBR was operated with a 6-hour cycle consisting of a slow-feeding phase (60 min), an anaerobic phase (20 min), an aerobic phase (180 min), and a sedimentation/discharge phase (100 min). Acetate was used as the sole carbon source. The SBR was operated at a hydraulic retention time (HRT) and a sludge retention time (SRT) of 12 h and 15 days, respectively. The pH was controlled at 7.00–7.50. The dissolved oxygen (DO) levels were maintained at 1.2–1.5 mg/L during the aerobic phase. The temperature was controlled at 25°C.

### DNA extraction, PCR amplification and sequencing

Genomic DNA was extracted from 26 activated sludge samples using the E.Z.N.A.® Soil DNA Kit (Omega Bio-tek, US) following the manufacturer’s instructions. V1-V3 and V4-V5 regions of the bacterial 16S rRNA gene were amplified with primer sets: 27F’ (5’-AGAGTTTGATCCTGGCTCAG-3′)-534R (5’-TTACCGCGGCTGCTGGCAC-3′) [39] and 515F (5’-GTGYCAGCMGCCGCGGTAA-3′)-926R (5’-CCGYCAATTYMTTTRAGTTT-3′) [47], respectively. High-throughput sequencing was performed. Detailed experimental steps are documented in Supplementary Text S2. The obtained data were deposited in the NCBI database under the BioProject No. PRJNA1046674.

## Results and discussion

### Coverage and specificity of FISH probes targeting PAOs and GAOs

FISH and related techniques are widely applied for *in-situ* identification and characterization of specific groups of PAOs and GAOs, where the coverage and specificity of the probes are keys to determining the accuracy and rationality of the obtained results. It is necessary to understand the strength and weaknesses of each probe commonly used for FISH analyses in the EBPR systems.

In EBPR systems, commonly found PAOs include *Ca*. Accumulibacter, *Tetrasphaera*, *Dechloromonas*, *Ca*. Phosphoribacter, *Ca*. Lutibacillus, and *Microlunatus phosphovorus.* Additionally, there are putative PAOs including *Tessaracoccus* and *Ca*. Obscuribacter [5, 13, 56, 57]. *Ca*. Competibacter, *Defluviicoccus,Ca*. Contendobacter, *Micropruina*, and *Propionivibrio* are commonly recognized GAOs [58, 59]. To enable targeted studies of these functional bacterial groups, specific probes defining each lineage were previously designed. This study focuses on the evaluation of probes for *Ca*. Accumulibacter, *Tetrasphaera, Dechloromonas,Ca*. Competibacter, and *Defluviicoccus*, as their respective probes are commonly used.

#### *Ca*. Accumulibacter

PAOmix (a mixture of PAO462, PAO651, and PAO846) is among the most widely used probe sets for *in-situ* identification and characterization of *Ca*. Accumulibacter [60].

In the SILVA database, among the 86 *Ca*. Accumulibacter reference sequences, 67 of them were original recovered from activated sludge. PAO846 showed the highest coverage (84.9%), followed by PAO651 (70.9%), and PAO462 (37.2%), of the total number (86) of reference sequences (unless otherwise specified). The resultant coverage of PAOmix (PAO846, PAO651 and PAO462) was 91.9%. Two of seven non-targeted *Ca*. Accumulibacter sequences by PAOmix were known to be retrieved from activated sludge (Fig. 1 and Table S2, see online supplementary material for a colour version of this table). Others were primarily recovered from soil, drinking water distribution systems, field-rice roots, or river water [61, 62] (Table S2, see online supplementary material for a colour version of this table). In addition, these PAO probes also covered outgroup members. PAOmix covers a total of 70 outgroup sequences. PAO651 was suggested as an alternative to overcome the weakness of PAOmix in the specificity [38]. PAO651 covered 12 non-*Ca*. Accumulibacter sequences, including 4 *Dechlorosoma* (accounting for 17.4% of *Dechlorosoma* reference sequences in the entire genus), 1 *Methyloglobulus*, 1 *Probionivibrio*, 1 *Ca*. Competibacter, and 1 *Thauera* reference sequences. PAO846 covered 37 non-*Ca*. Accumulibacter sequences, including eight *Propionivibrio* (accounting for 10.5% of all *Propionivibrio* sequences in the reference database), two *Dechlorosoma*, four uncultured Cyanobacteriales, one *Dechlorobacter*, and one *Ferribacterium* sequences. PAO462 covered 11 non-*Ca*. Accumulibacter sequences (Fig. 1), including five *Propionivibrio* (accounting for 26.0% of all *Propionivibrio* reference sequences), four *Dechlorosoma* (accounting for 17.4% of all *Dechlorosoma* reference sequences), and one GKS98 freshwater group sequences.

Apart from SILVA, MiDAS is a comprehensive and specific reference database for microbes in activated sludge [63]. In the MiDAS database, the coverages of PAO846, PAO462, and PAO651 on *Ca*. Accumulibacter-affiliated sequences (218 in total) were 96.3%, 47.2 and 85.8%, respectively (Table 1, Fig. 1), with a combined coverage of 96.8%. For outgroup members (i.e. non-*Ca*. Accumulibacter), PAO462 and PAO846 covered *Propionivibrio* sequences only (24 and 34, respectively). PAO651 covered four Sumerlaeaceae family sequences (three midas_g_5270 and one midas_g_82290 members).

All these three probes showed higher coverage of confirmed *Ca*. Accumulibacter sequences in the MiDAS database than in the SILVA database. The use of hybrid probes enables the highest coverage but the highest number of non-targets. The presence of *Ferribacterium*, *Dechlorosoma*, *Ca*. Competibacter, *Thauera*, and *Propionivibrio* that frequently found in WWTPs could result in false positive results for PAOmix-based FISH detection of *Ca*. Accumulibacter. For instance, in the SILVA database, *Ca*. Competibacter KF697440, which was recovered from a membrane bioreactor, is covered by PAO651 [64]. Additionally, PAOmix covers a large number of *Propionivibrio* sequences (10.5%). Albertsen *et al.* [38] showed high occurrences of *Propionivibrio* as novel GAOs in lab- and full-scale EBPR systems, which interfered with FISH detection of *Ca*. Accumulibacter. FISH quantification using PAO651 yielded 5–15% lower relative abundance than using PAOmix. A majority of the missed fraction was *Propionivibrio* (5.3% coverage), which could be targeted by Prop207 [3, 38, 65]. Noteworthy, PAO846 covered four Cyanobacteriales sequences in the SILVA database. Coincidentally, Ji *et al*. [66] reported that in a microalgal-bacterial granular sludge system, the dominant microalgae, i.e. *Pantanalinema-*related members (belonging to Cyanobacteriales) were capable of poly-P accumulation and P cycling, showing a PAO-phenotype. The microalgae were successfully hybridized by PAOmix, showing that PAO846 did cover Cyanobacteriales members.

In addition to PAOmix, probes targeting different clades of *Ca*. Accumulibacter were previously designed (Table 1), i.e. Acc444, HAcc466 and HAcc426 for clade ІA; Acc184, CAcc184 and Acc623 for Clade IIC. Acc119, HAcc99, HAcc139, and CAcc119 for Clade IIA members [67, 68]. The coverage of these clade-specific probes on *Ca*. Accumulibacter-affiliated sequences are shown in Table 1. Most recently, a set of species-level FISH probes were designed to resolve *Ca*. Accumulibacter species with different morphologies, including Acc469, Acc471, Acc1011, Acc635, Acc470, and Acc471_2 (Table 1). In the SILVA database, Acc184, CAcc184, Acc469, Acc1011, Acc635, Acc470, Acc471_2, and Acc623 showed 100% specificity. Acc184 (JQ726366), HAcc426 (GQ389158), CAcc119 (JQ726366, KJ807957), and Acc471_2 (JQ726366) covered *Ca*. Accumulibacter sequences which was not covered by the commonly used probe set PAOmix. In the MiDAS database, Acc184, CAcc184, Acc469, Acc1011, Acc635, Acc470, Acc471_2, and Acc623 showed 100% specificity. Via our analysis, it seems that a combined use of PAO651 with Acc471 and Acc184 would confer an improved coverage of sequences in the SILVA database (from 70.9% to 81.4%) of *Ca*. Accumulibacter without notably compromising the specificity (the number of outgroup sequences increased from 12 to 13). And, in the MiDAS database, the coverage would improve from 85.8% to 91.7% with no loss in the specificity. Additional information about the coverage and specificity of probes for different *Ca*. Accumulibacter Clades is shown in Text S3. Collectively, in the SILVA database, there are 3 (3.5%) *Ca*. Accumulibacter reference sequences (all of them are deeply-branched Clade I members, Fig. 1 and Table S2, see online supplementary material for a colour version of this table) which are not covered by any existing probes. None of them was originally recovered from activated sludge. In the MiDAS database (which is specific for microbes in activated sludge), there are 3 (1.4%) *Ca*. Accumulibacter sequences (all belong to Clade II, Fig. S1; the identities of these sequences are given in Table S3, see online supplementary material for a colour version of this figure), which are not covered by any existing probes, indicating a potential space for further improvements in FISH probe design for *Ca*. Accumulibacter detection. The abundances of these *Ca*. Accumulibacter in lab- and full-scale EBPR systems also deserve further investigation.

#### Tetrasphaera

*Tetrasphaera* was primarily isolated using modified cell extract agar incorporating 0.25% Casamino acids [69]. This group of bacteria was later identified as PAOs as indicated by 4,6-diamino-phenylindole staining [23]. Different from *Ca*. Accumulibacter, *Tetrasphaera* were shown capable of taking up complex organics such as amino acids and glucose without PHA storage, and surviving anaerobic conditions via fermentation [8, 70-72]. They were considered to be more advantageous for P removal from wastewater with low VFA contents [8]. There are three clades in the *Tetrasphaera* lineage. Clade 1 members (branched rods and cocci in tetrads) were reported to be incapable of acetate usage. Clade 2 members (cluster of tetrads, rods, and/or filaments) do not seem to take up glucose and casamino acid [25]. A recent study performed by Close *et al*. [73] showed that a highly enriched *Tetrasphaera* culture (95% biovolume by FISH quantification) predominated by Clade 2 members were able to store and circulate PHA (PHV exclusive) with Casein or an amino acid mixture as carbon sources. Clade 3 members (cluster of tetrads, rods, and/or small cocci) were described as competitive users of casamino acid, glutamic acid, and glucose [74]. Recently, *Ca*. Phosphoribacter and *Ca*. Lutibacillus which were previously recognized as Clade 3 *Tetrasphaera* are redefined as two novel PAO genera. They were found to have a higher ability to use fructose than *Tetrasphaera* [26]. New FISH probes were designed to cover these two genera (Phos741, Phos601, and Luti617) [24, 26]. *Ca*. Phosphoribacter- and *Ca*. Lutibacillus-related sequences are excluded from the collection of *Tetrasphaera* reference sequences when evaluating the performances of *Tetrasphaera*-targeting probes.

Actino-1011 was primarily designed to target *Tetrasphaera japonica*-related members which showed high relative abundance in WWTPs as PAOs [23]. Actino-221 and Actino-658 were then developed to further distinguish different morphotypes which were targeted by Actino-1011. Actino-221 covers cocci *Tetrasphaera*. Actino-658 (it was recently found to mainly target *Ca*. Phosphoribacter, thus became an *Ca*. Phosphoribacter specific probe [26]) covers rod ones [24]. Subsequent research showed that Clade 1 and a large portion of Clade 2 members were not covered by these two probes [25], resulting in significantly underestimated diversity of *Tetrasphaera* in full-scale EBPR plants. Elo-1250, Tet1–266, Tet2–87, Tet2–174, Tet2–823, Tet2–831, Tet2–842, Tet2–892, Tet3–19, and Tet3–654 were thus designed to target the diverse *Tetrasphaera* which was not covered by the Actino probes [25]. Additionally, Tetra732 and Tetra67 were designed to co-target *Tetrasphaera* species midas_s_299 which was found as a top 10 most abundant *Tetrasphaera* species in global WWTPs [26]. Tetra183 was designed to cover *Tetrasphaera*-related genera [26].

**Figure 2:**
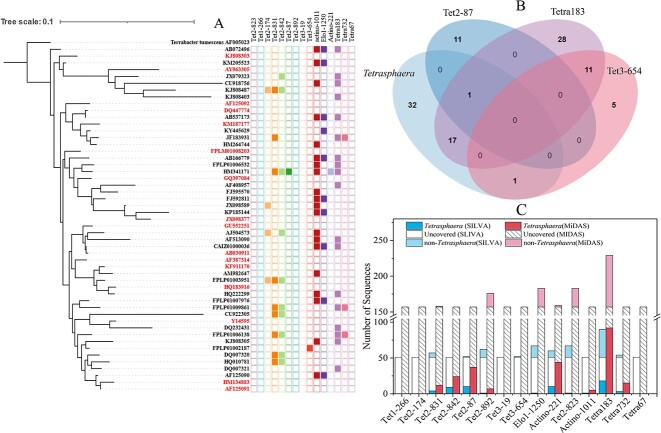
Coverage of commonly used FISH probes for *Tetrasphaera*. A. A phylogenetic tree of *Tetrasphaera* showing the coverage of each probe. The tree was built using refined reference sequences obtained from the SILVA database (figure S1). Not all the sequences were originally recovered from activated sludge. Color parts of the outer circle indicates the sequences that were covered by each probe. The sequences which were not covered by exsiting probes were marked in different font colors (detailed origination information of these sequences is given in Table S3). The maximum likelihood method was used with the Tamura-Nei model and none branch swap fitter for tree construction. The scale bar represents substitutions per nucleotide base. B. A Venn diagram showing the covered and/or co-covered number of *Tetrasphaera* sequences (with refined reference sequences obtained from the SILVA database) by each probe. C. The numbers of target and nontergeted sequences which were covered by each probe in the SILVA and the MiDAS databases.

In the SILVA database, the coverage and specificity of these probes towards 51 *Tetrasphaera*-affiliated sequences (15 of them were originally recovered from activated sludge or WWTPs) were evaluated (Table 1, Fig. 2). For the Actino probes, Actino-1011 showed the highest coverage (39.2%), however, with low specificity. It covered 170 non-*Tetrasphaera* sequences, including 11 *Lapillicoccus* (accounting for 59.7% of all *Lapillicoccus* reference sequences), 14 unidentified Intrasporangiaceae (66.7%), 14 *Ornithinicoccus* (77.8%), 8 *Aquipuribacter* (88.9%), and 27 *Knoellia* sequences (77.1%), etc. Low coverage was observed for Actino-221 (2.0%, only 1 *Tetraphaera* sequence) with no coverage of non-*Tetraspheara* sequence. Elo-1250, which was designed to target *Tetraspheara elongata-*related members, showed a coverage of 19.6%. It covered nine non-*Tetrasphaera* sequences (Table S4, see online supplementary material for a colour version of this figure). For the Tet probes, Tet2–842 showed the highest coverage (19.6%), followed by Tet2–831 (17.6%). Both probes showed high specificity, 100% for Tet2–831. Tet2–842 covered 1 non*-Tetrasphaera* sequence (*Mobilicoccus* KX056042). Other Tet probes all showed coverage values below 10% (7.8%, 0.0%, 2.0%, 0.0%, 0.0% and 2.0% for Tet2–174, Tet3–19, Tet2–87, Tet1–266, Tet2–892, and Tet3–654, respectively), probably because these probes were primarily designed for different *Tetrasphaera* clade members. Tet2–174 and Tet2–87 covered 6 (3 *Lapillicoccus* and 3 unidentified Intrasporangiaceae) and 11 (3 *Propionicicella*, 1 *Propionicimonas*, and 7 PeM15) non-*Tatraspheara* sequences, respectively (Table S4, see online supplementary material for a colour version of this figure.). Tetra732 covered one uncultured Intrasporangiaceae, one *Huakuichenia* and one *Marihabitans* sequence, Tetra183 covered three *Lapillicoccus* and three uncultured Intrasporangiaceae, two PeM15, five uncultured Micrococcales sequences, and one each sequence from *Janibacter*, uncultured Synergistaceae, *Sedimentibacter*, *Dietzia*, *Pedococcus-Phycicoccus*, and RBG-13-54-9, respectively. Tetra67 covered five uncultured Intrasporangiaceae, two *Lysinimicrobium*, two *Demequina* sequences, and one each sequence from *Actinotalea*, *Sanguibacter-Flavimobili*, *Jatrophihabitan*, PeM15, *Dietzia*, and DTU014.

In the MiDAS database (157 *Tetrasphaera* sequences in total), the coverages of these probes are generally comparable to those in the SILVA database (Table 1). As for their specificities, Tet2–174 covered 1 *Janibacter* sequence. Tet2–87 covered 18 midas_g_957 and 1 *Propionicimonas*. Elo1–1250 covered one midas_g_3069 and one midas_g_99 sequences. Actino1011 covered five midas_g_99, one *Knoellia*, one *Ornithinibacter*, and five *Propionicicella* sequences. Tetra183 covered one *Pedococcus-Phycicoccus* and one midas_g_99 sequences. Tetra67 covered three *Demequina*, four *Lysinimicrobium*, two *Actinotalea*, and two *Lapillicoccus* sequences. Tet2–831, Tet2–842, Actino221, and Tetra732 showed 100% specificities in the MiDAS.

Overall, there were 27.5% (14 sequences) and 14.6% (23 sequences) *Tetraspheara* sequences in the SILVA and the MiDAS databases, respectively, which were not covered by existing FISH probes. The low coverage of existing probes and their shortage in specificities may result in incorrect understandings of the true relative abundance of *Tetraspheara* with FISH. Probes with improved specificity and coverage are needed. Comprehensively considering, Elo1–1250 and Tet2–842 may be used together to confer the highest coverage of *Tetrasphaera* (combined coverage of 39.2% and 51.5%) with nine and two outgroup sequences, respectively, in the SILVA and MiDAS databases.

#### *Ca*. Phosphoribacter and *Ca*. Lutibacillus

Actino-658, Phos741, and Phos601 were designed for *Ca*. Phosphoribacter. Luti617 was designed for *Ca*. Lutibacillus (Figure S1B, see online supplementary material for a colour version of this figure). In the SILVA database (Table 1), Actino-658, Phos741, and Phos601 covered 15, 6, and 6 *Ca*. Phosphoribacter sequences (28 in total), respectively. Luti617 covered 3 *Ca*. Lutibacillus sequences (5 in total). In the MiDAS database (Table 1), Actino-658, Phos741, and Phos601 covered 26, 38, and 7 *Ca*. Phosphoribacter sequences (74 in total), respectively. Luti617 covered 5 *Ca*. Lutibacillus sequences (15 in total). As for the specificity of these probes, in the SILVA database, Actino-658 covered 1 *Tetrasphaera* and 1 PeM15 sequences. Phos741 covered 2 *Tetrasphaera* and 1 *Ornithinicoccus* sequences. In contrast, in the MiDAS database, all these probes showed an 100% specificity, except for Phos741, which covered 1 *Tetrasphaera* sequence*.*

#### Dechloromonas

Certain *Dechloromonas* members were considered as putative PAOs [75]. Some others were suspected as GAOs [33, 76]. Recently, two metagenome-defined *Dechloromonas* (i.e. *Ca*. *Dechloromonas* phosphoritropha and *Ca*. *Dechloromonas* phosphorivorans) were confirmed for the first time as PAOs [75], although with the roles of other members in this genus yet to be completely determined. FISH probes used to target *Dechloromonas* include DEMFE455, DCMAG455, Bet135, Dech443, and Dech453 [68] (Table 1).

In the SILVA database, the coverages of these probes were low (2.8%–22.1%) (Table 1). Of the 181 *Dechloromonas* reference sequences, 84 were originally recovered from activated sludge. 64.1% of them were not covered by any existing probes (listed in Table S5, see online supplementary material for a colour version of this table). In addition, except for Dech443, all probes covered non-*Dechloromonas* sequences (6, 48, 35, and 2 for DCMAG455, DEMFE455, Bet135, and Dech453, respectively). DCMAG455 covered one uncultured Rhodocyclaceae, four *Ferribacterium*, and one *Quatrionicoccus* sequences. DEMFE455 covered 12 uncultured Rhodocyclaceae, 14 *Ferribacterium*, 3 *Dechlorobacter*, 1 *Dechlorosoma*, and 2 *Ca*. Accumulibacter sequences. Bet135 covered 1 *Ferribacterium*, 2 *Dechlorobacter*, 1 *Dechlorosoma*, 1 *Ca*. Accumulibacter, 3 *Quatrionicoccus,* and 1 *Thauera* sequence. Dech453 covered one MND1 and one Hydrogenophilaceae sequences.

**Figure 3:**
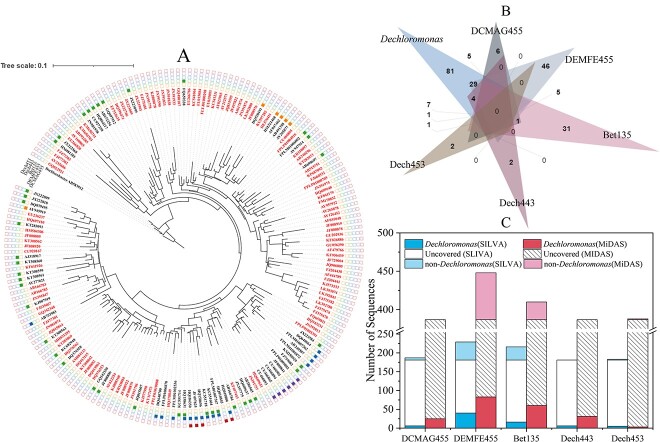
Coverage of commonly used FISH probes for *Dechloromonas*. A. A phylogenetic tree of *Dechloromonas* showing the coverage of each probe. The tree was built using reference sequences obtained from the SILVA database. Not all the sequences were originally recovered from activated sludge. Color parts of the outer circle indicate the sequences that were covered by each probe. The sequences which were not covered by exsiting probes were marked in red (detailed origination information of these sequences is given in Table S4). The Maximum Likelihood method was used with the Tamura-Nei model and none branch swap fitter for tree construction. The scale bar represents substitutions per nucleotide base. B. A Venn diagram showing the covered and/or co-covered number of *Dechloromonas* sequences (reference sequences obtained from the SILVA database) by each probe. C. The numbers of target and nontergeted sequences which were covered by each probe in the SILVA and the MiDAS databases.

In the MiDAS database, among 387 *Dechloromonas* reference sequences, the coverages of these probes were 0.8%–21.4% (Table 1). Dech443 again showed 100% specificity. DEMFE455 covered 61 non-*Dechloromonas* sequences, including 38 *Ferribacterium*, 11 midas_g_94, 4 *Nitrotoga*, 3 midas_g_913, 2 *Ca*. Accumulibacter, 2 midas_g_1040, and 1 *Dechlorobacter* (Fig. 3). Bet135 covered 23 non-*Dechloromonas* sequences, including 7 midas_g_94, 6 midas_g_168, 5 Z-35, 4 *Quatrionicoccus*, and 1 midas_g_4927 sequences. Dech453 covered 1 *Ca*. Accumulibacter sequence (FLASV28694) beyond *Dechloromonas*.

Noteworthily, DEMEF455, Bet135, and Dech453 covered *Ca*. Accumulibacter sequences. DEMEF455 covered 2 *Ca*. Accumulibacter sequences in the SILVA (FQ659038 and FQ658970) database and 1 in the MiDAS (FLASV61645) database. Dech453 covered 1 *Ca*. Accumulibacter (FLASV28694) in the MiDAS database. Bet135 covered 1 *Ca*. Accumulibacter (FPLP01009890) in the SILVA database. Special attention is needed when using these probes for the characterization and determination of the PAO/GAO nature of their defined *Dechloromonas*. Among them, *Ca*. Accumulibacter sequences FQ659038, and FQ658970 were not covered by any *Ca*. Accumulibacter probes. FPLP01009890 was covered by HAcc139. Interestingly, Kong *et al*. observed ~30% overlap between Bet135 and PAOmix hybridized cocci-shaped cells which were capable of P cycling, although, in our evaluation, no *Ca*. Accumulibacter or *Dechloromonas* sequence was co-targeted by Bet135 and PAOmix. The result implied that there might be unidentified PAOs in their co-covered genera: *Ferribacterium*, *Dechlorobacter*, and/or *Dechlorosoma*.

#### *Ca*. Competibacter

GAOs are another group of bacteria of interest in the EBPR system. Their occurrence in EBPR systems is typically undesirable since they compete with PAOs for organic carbon without contributing to P removal [11, 27]. *Ca*. Competibacter is a commonly observed genus of GAOs in lab- and full-scale EBPR systems. GAO431 (GAOQ431) and GAO989 (GAOQ989) have been widely used to target this group of microorganisms.

**Figure 4:**
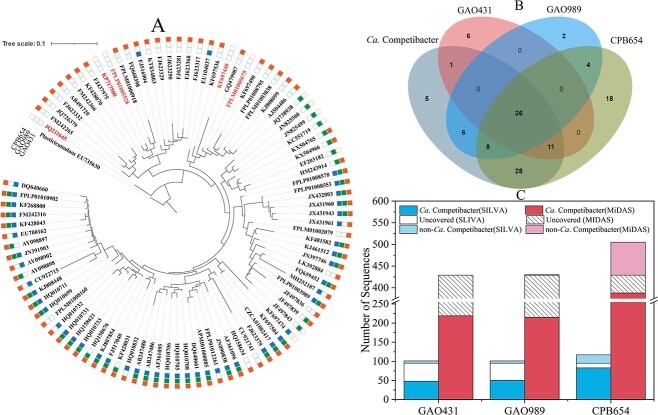
Coverage of commonly used FISH probes for *Ca*. *Competibacter*. A. A phylogenetic tree of *Ca*. Competibacter showing the coverage of each probe. The tree was built using reference sequences obtained from the SILVA database. Not all the sequences were originally recovered from activated sludge. Color parts of the outer circle indicate the sequences that were covered by each probe. The sequences which were not covered by exsiting probes were marked in different font colors (detailed origination information of these sequences is given in Table S5). The maximum likelihood method was used with the Tamura-Nei model and none branch swap fitter for tree construction. The scale bar represents substitutions per nucleotide base. B. A Venn diagram showing the covered and/or co-covered number of *Ca*. Competibacter sequences (reference sequences obtained from the SILVA database) by each probe. C. The numbers of target and nontergeted sequences which were covered by each probe in the SILVA and the MiDAS databases.

In the SILVA database, among the 95 *Ca*. Competibacter reference sequences (56 of them were recovered from activated sludge), the coverage of GAO431 and GAO989 were 50.5% and 52.6%, respectively, with a combined value of 65.3% (Fig. 4). Thirty-three *Ca*. Competibacter sequences (19 of them were recovery from activated sludge) were not covered by GAO431 or GAO989. Additionally, these two probes each covered six non-*Ca*. Competibacter sequences, three *Aquicella,* two uncultured Diplorickettsiaceae, and one BD1–7 clade sequences for GAO431, four *Ca*. Contendobacter, one *Sulfurifustis*, and one *Desulfobulbus* sequences for GAO989. For improved coverage and specificity, CPB-654 was designed [27], the coverage of which reached 87.4%, with 12 non-covered *Ca*. Competibacter sequences (mainly distributed in Clade CS1). Additionally, this probe covered 22 non-*Ca*. Competibacter sequences, all of which belong to *Ca*. Contendobacter (representing all *Ca*. Contendobacter reference sequences in the SILVA database, except AY098909). Given the role of *Ca*. Contendobacter as GAOs in EBPR systems, CPB-654 is a promising probe for the detection of *Ca*. Competibacteraceae family members, covering 105 (83 *Ca*. Competibacter and 22 *Ca*. Contendobacter) out of 118 (95 *Ca*. Competibacter and 23 *Ca*. Contendobacter in total) *Ca*. Competibacteraceae references sequences, with an overall coverage of 89.0% and a specificity of 100%. There were five *Ca*. Competibacter and one *Ca*. Contendobacter reference sequences were not covered by any existing probes, among which 2 *Ca*. Competibacter and 0 *Ca*. Contendobacter sequences were recovered from activated sludge (Table S6,see online supplementary material for a colour version of this table).

In the MiDAS database, the coverage of GAO431, GAO989, and CPB654 for *Ca*. Competibacter (429 in total) were 50%, 50.1%, and 90.4%, respectively, with a combined coverage value of 90.4% (i.e. CPB654 covered all GAO989- or GAO431-targeted *Ca*. Competibacter sequences). GAO431 showed 100% specificity. GAO989 and CPB654 covered 1 and 74 *Ca*. Contendobacter sequences, respectively. Additionally, CPB654 covered two non-*Ca*. Competibacter and non-*Ca*. Condentobacter sequences (belonging to midas_g_77434). Overall, GAO431, GAO989 and CPB654 achieved 90.4% and 95.7% coverages for *Ca*. Competibacter and *Ca*. Contendobacter, respectively. Again, CPB-654 is an excellent probe for the *Ca*. Competibacteraceae family, although there were 41 *Ca*. Competibacter sequences were not covered by any existing probes (Table S3, see online supplementary material for a colour version of this table). For combined detection and analysis of *Ca*. Competibacter and *Ca*. Contendobacter, CPB654 is desirable. For *Ca*. Competibacter alone, GAO989 + GAO431 would be a rational choice.

#### Defluviicoccus

*Defluviicoccus* are another group of GAOs commonly found in lab- and full-scale EBPR systems [13, 16, 77], which was primarily described by Maszenan *et al*. [19], occurring in tetrads of cocci in a brewery WWTP. There are four clusters in this genus. Cluster I, II, and IV members appeared as cocci in tetrads or cells in clumps. Cluster III members were shown to have a “*Nostocoida limicola*-like” filamentous morphology [78]. The filamentous Cluster III members were reported to commonly occur in full-scale WWTPs and may cause sludge bulking [79]. Recent studies suggested that Cluster III *Defluviicoccus* may be different in carbon uptake bioenergetics from their Cluster I and II relatives, which may have allowed them to coexist with *Ca*. Accumulibacter and *Ca*. Competibacter in EBPR systems under controlled carbon source supply conditions [11, 80].

In the SILVA database, the coverages of commonly used *Defluviicoccus* probes (towards 75 reference sequences, among which 17 was originally recovered from activated sludge) were as follow: 9.3%, 5.3%, and 5.3% for Cluster I probes TFO-DF218, TFO-DF618, TFO-DF862; 9.3% and 10.7% for Cluster II probes DF1020 and DF988; 1.3%, 1.3%, and 4.0% for Cluster III probes DF1004, DF1013; and 4.0%, 1.3%, and 4% for Cluster IV probes DF198, DF181A, and DF181B, respectively (Table 1). All *Defluviicoccus* sequences which were targeted by DF1020 were covered by DF988. The resultant combined coverage of these probes was 29.4%.

**Figure 5:**
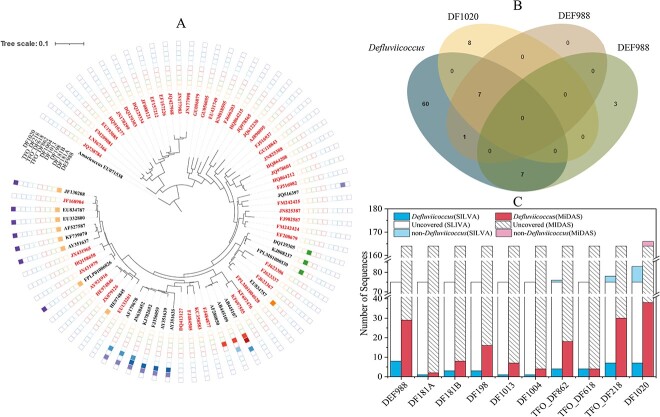
Coverage of commonly used FISH probes for *Defluviicoccus*. A. A phylogenetic tree of *Defluviicoccus* showing the coverage of each probe. Color parts of the outer circle indicates the sequences that were covered by each probe. The tree was built using reference sequences obtained from the SILVA database. Not all the sequences were originally recovered from activated sludge. The sequences which were not covered by existing probes are marked in different font colors (detailed origination information of these sequences is given in Table S6). The maximum likelihood method was used with the Tamura-Nei model and none branch swap fitter for tree construction. The scale bar represents substitutions per nucleotide base. B. A Venn diagram showing the covered and/or co-covered number of *Defluviicoccus* sequences (reference sequences obtained from the SILVA database) by each probe. C. The numbers of target and nontergeted sequences which were covered by each probe in the SILVA and the MiDAS databases.

In the MiDAS database, these probes showed 18.3% (TFO-DF218), 2.4% (TFO-DF618), 11% (TFO-DF862), 23.2% (DF1020), 17.7% (DF988), 2.4% (DF1004), 4.3% (DF1013), 9.8% (DF198), 1.2% (DF181A), 4.9% (DF181B) coverage of *Defluviicoccus* sequences (164 in total), with a combined coverage of 56.7% (Fig. 5).

Despite the low coverage of these probes, their specificities are overall high, with only DF862, DF218, and DF1020 covering one (uncultured Defluviicoccales), three (uncultured Thalassobaculales), and eight (one A714019, one *Ca*. Jidaibacter, one *Ca*. Riegeria, two *Omnitrophales*, one DEV007, and two WCHB1–41) non-*Defluviicoccus* sequences in the SILVA database. In the MiDAS database, only DF1020 covered non-*Defluviicoccus* sequences (one midas_g_77531 and one midas_g_49838). However, because of the large proportion (65.6% and 43.3% in the SILVA and MiDAS databases, respectively) of *Defluviicoccus* sequences were not covered by any existing probes, there are still large rooms in the design of novel FISH probes for improved understandings of their ecophysiology in EBPR systems.

#### Overview

In summary, there are a number of sequences that were not covered by commonly used probes for each PAO and GAO groups. Almost all probes covered non-target sequences, suggesting potential discrepancies between the FISH results and the actual community composition (depending on which species was present and/or dominated in the community). A systematic location of sequences that are not covered by existing probes is important for the understanding of the blind spot of FISH and related analyses, benefiting researches on the hidden microbial community in these known PAO and GAO lineages in EBPR systems. To facilitate future research, we have sorted out these sequences which are documented in Supplementary Tables S2-S7, see online supplementary material for a colour version of these tables. Since SILVA is a comprehensive database, it is necessary to further sort out the sequences which were recovered or had occurred in wastewater treatment systems. Overall, among these sequences, 0 *Ca*. Accumulibacter, 6 *Tetrasphaera*, 55 *Dechloromonas*, 2 *Ca*. Competibacter, and 8 *Defluviicoccus* sequences were observed to have occurred in wastewater treatment systems (Text S4 and Table S2–S7, see online supplementary material for a colour version of these tables). As for the MiDAS database, since it is essentially a database for microbes in activated sludge, the sequences which were not covered by any existing probes in the MiDAS database would all potentially be relevant to the EBPR system (including 3 *Ca*. Accumulibacter, 0 *Tetrasphaera*, 209 *Dechloromonas*, 41 *Ca*. Competibacter, and 71 *Defluviicoccus* sequences, Text S4 and Table S3, see online supplementary material for a colour version of these table).

Additionally, although most FISH probes are not 100% specific, it does not necessarily mean that these probes are not effective. Via combined usage of multiple biomolecular techniques (e.g. 16S rRNA gene amplicon sequencing or metagenomics), and the selection of appropriate probes based on known community compositions, the advantages of FISH analysis may be maximized with minimized and controllable biases. The analyses performed in this study may also facilitate the selection of probes when FISH was used together with sequencing-based techniques.

### Primer coverage evaluation in PCR amplifying

**Figure 6:**
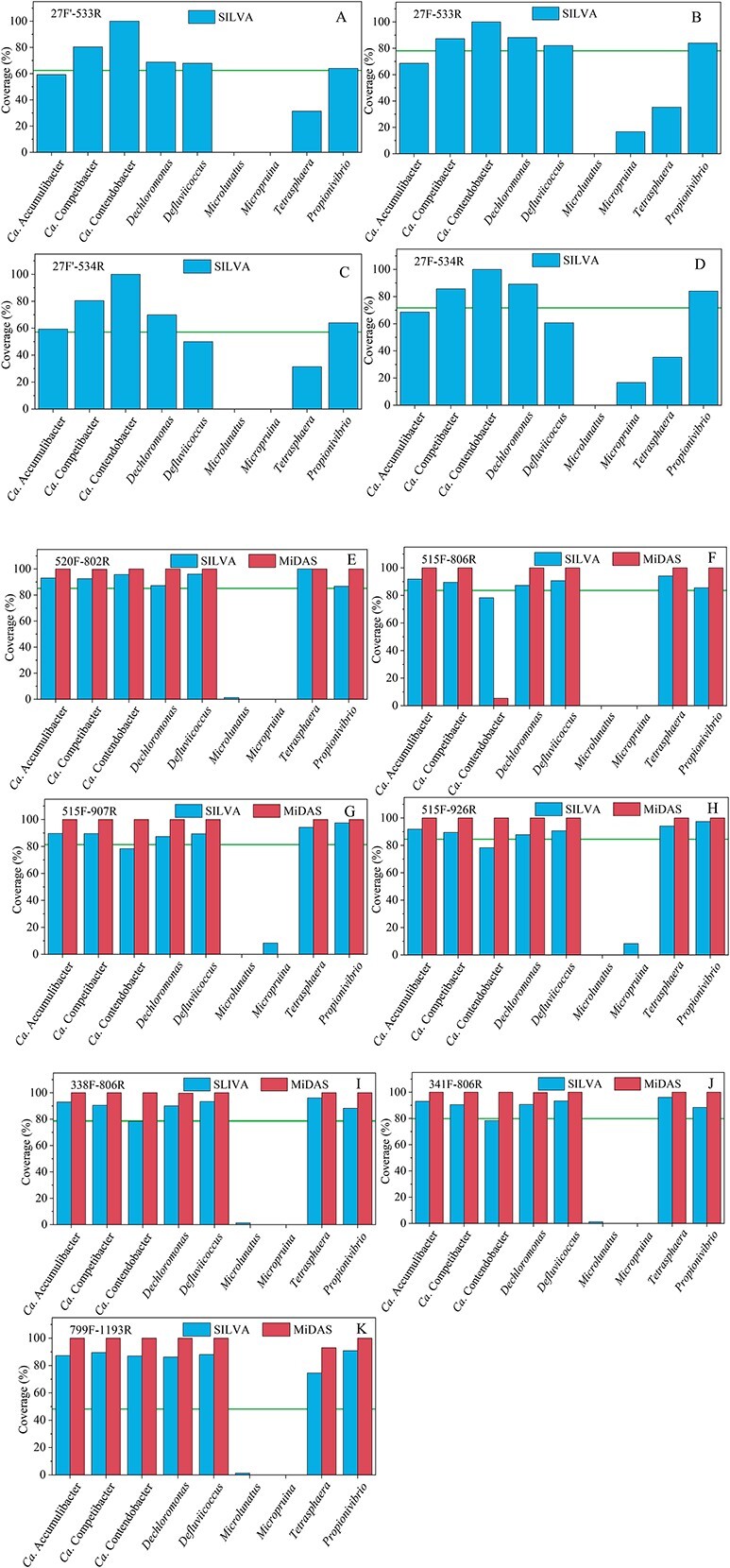
Coverage of commonly used 16 s rRNA gene amplicon primer sets on known polyphosphate accumulating organisms (PAOs) and glycogen accumulating organisms. A. 27F'-533R; B. 27F-533R; C. 27F'-534R; D. 27F-534R; E. 520F-802R; F. 515F-806R; G. 515F-907R; H. 515F-926R; I. 338F-806R; J. 341F-806R; K. 799F-1193R. The coverage of each primer set on total bacteria was indicated as a line in each panel.

In addition to FISH, PCR amplification-based methods (e.g. 16S rRNA gene amplicon sequencing) are widely applied for bacteria community analysis and characterization in EBPR systems. The coverages of commonly used universal primer sets (Table S1, see online supplementary material for a colour version of this table) on known PAO and GAO groups were analyzed (Fig. 6).

27F-533R and 27F-534R are commonly used to target the V1-V3 region of the bacterial 16S rRNA gene. In the SILVA database, compared to 27F-533R, 27F-534R showed slightly lower coverage of *Defluviicoccus* (by 21.4%) and *Ca*. Competibacter (by 1.8%). There was no significant difference in the coverage of *Ca*. Accumulibacter, *Tetrasphaera*, *Ca*. Phosphoribacter, *Ca*. Lutibacillus, *Micropruina* and *Propionivibrio* by 27F-533R and 27F-534R (the specific coverage value on each taxon is documented in Table S1, see online supplementary material for a colour version of this table, and Fig. 6). In the MiDAS database, the coverage of these two primer sets was not evaluated since the 27F-end primer sequences were clipped for all sequences.

338F-806R and 341F-806R are two primer sets targeting the V3-V4 region. The coverages of these two primers sets are overall similar in both databases, both achieving >90% coverage for EBPR related taxa (except that 78.3% for *Ca*. Contendobacter) in the SILVA database and nearly 100% in the MiDAS database (Detailed in Table S1, See online supplementary material for a colour version of this table, and Fig. 6). For *Ca*. Phosphoribacter and *Ca*. Lutibacillus, 338F-806R covered 22 and 5, 341F-806R covered 23 and 5 out of 28 and 5 sequences, respectively, in the SILVA database. These two primer sets covered all known *Ca*. Phosphoribacter (74 in total) and *Ca*. Lutibacillus (15 in total) sequences in the MiDAS database except for *Ca*. Phosphoribacter FLASV50698.

515F-806R and 520F-802R are two primer sets used to target the V4 region. In the SILVA database, 515F-806R showed slightly lower coverage values than 520F-802R for *Ca*. Accumulibacter, *Ca*. Contendobacter, *Defluviicoccus*, *Tetrasphaera*, and *Ca*. Competibacter; and the same coverage for *Dechloromonas*, *Ca*. Phosphoribacter and *Ca*. Lutibacillus (Detailed in Table S1, see online supplementary material for a colour version of this table, and Fig. 6)*.* In the MiDAS database, the coverages of 520F-802R were 100% for *Ca*. Accumulibacter, *Ca*. Contendobacter, *Dechloromonas*, *Defluviicoccus*, *Ca*. Phosphoribacter, *Ca*. Lutibacillus, and *Tetrasphaera*, and 99.77% for *Ca*. Competibacter. 515F-806R showed 100% coverage for all the above-mentioned PAOs and GAOs in the MiDAS database, except for an extremely low coverage of *Ca*. Contendobacter (5.4%). Generally, 520F-802R showed overall higher coverages for PAOs and GAOs than 515F-806R.

515F-907R and 515F-926R are two primer sets targeting the V4–5 region. In the SILVA database, the coverages of 515F-907R and 515F-926R in the SILVA database for PAOs and GAOs in this study were 87.3%–94.1%. The lowest coverage was found for *Ca*. Contendobacter (both at 78.3%). Both primer sets covered 25 *Ca*. Phosphoribacter (28 in total) and 5 *Ca*. Lutibacillus (5 in total) sequences. Both primer sets showed 100% coverages for these PAOs and GAOs in the MiDAS database. Overall, 515F-926R showed slightly higher coverage than 515F-907R (especially for Ca. Accumulibacter, 91.9% and 90.0%, respectively), although the differences were minor.

For each specific PAO and GAO group in the SILVA database, primer sets showing the highest coverages were 338F/341F-806R and 520F-802R for *Ca*. Accumulibacter (93.0%), 338F/341F-806R for *Tetrasphaera* (96.1%), 341F-806R for *Dechloromonas* (90.6%), 338F/341F-806R for *Ca*. Competibacter (90.5%), 520F-802R for *Ca*. Contendobacter (95.7%), 520F-802R for *Defluviicoccus* (96.0%), and 515F-907R/926R for *Propionivibrio* (97.4%). In addition, all these primer sets showed extremely low coverage (0%–36%) for *Microlunatus*. *M. phosphovorus* was shown as a PAO capable of glucose and amino acids usage for EBPR [81], the intracellular P content of which was reported to reach 10% [81]. The extremely low coverages of commonly used primer set on *Microlunatus* implied that the occurrences and roles of *Microlunatus* might have been significantly overlooked. New primer sets or FISH probes are required for the capture and identification of *Microlunatus-*related microorganisms in EBPR systems.

Apart from *Microlunatus*, there are two reference sequences which were not covered by any of these primer sets (i.e. *Dechloromonas* AB240296 and *Defluviicoccus* JN178299) in the SILVA database. All reference sequences in the MiDAS database were covered by at least one primer set.

Noteworthily, 27F has a non-degenerated version (i.e. AGAGTTTGATCCTGGCTCAG, denoted as 27’F). In studies, the non-degenerated 27F was used in combination with 533R or 534R for 16S rRNA gene amplicon sequencing. The coverage of 27F and 27’F was further evaluated when they were used together with 534R. In the SILVA database, 27F’ showed significantly lower coverages. i.e. 85.7% and 80.4% for *Ca*. Competibacter, 89.2% and 69.9% for *Dechloromonas*, 60.5% and 50.0% for *Defluviicoccus*, for 27F-534R and 27F’-534R, respectively. But both primer sets have the same coverage for *Ca*. Accumulibacter (61.6%) and *Tetrasphaera* (31.4%). The use of degenerated 27F instead of 27’F is thus preferable for 16S rRNA amplicon sequencing and related analyses (Figure S2, see online supplementary material for a colour version of this figure).

Above all, 520F-802R and 341F-806R seemed to be the most preferable primer sets for EBPR community analyses. Both primer sets achieved more than 85% coverage for major PAOs and GAOs in the SILVA database (92.7% and 91.1% for *Ca*. Accumulibacter, 95.5% and 93.3% *Tetrasphaera,* 87.3% and 90.6% for *Dechloromonas,* 92.6% and 88.8% for *Ca*. Competibacter, and 96.0% and 93.3% for *Defluviicoccus* for 520F-802R and 341F-806R, respectively), benefiting an improved capturing of these functional groups.

### Differences in community analysis results aroused by primer selection and database annotations

**Figure 7:**
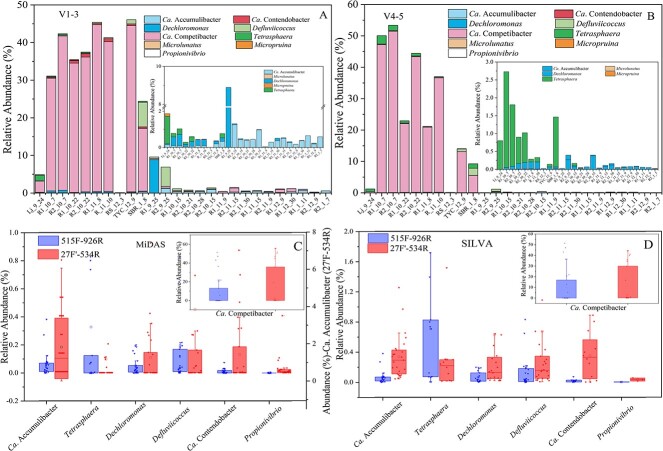
Effects of primer set and annotation database selection on the 16S rRNA gene amplicon analyses of enhanced biological phosphorus removal community. A. Polyphosphate accumulating organism (PAO) and glycogen accumulating organism (GAO) community composition obtained with 27F’-534R. B. PAO and GAO community composition obtained with 515F-926R. C. Distribution of relative abundances of known PAOs and GAOs as indicated by 16S rRNA gene amplicon analysis with different primer sets annotated using the SILVA 138 refNR database. D. Distribution of relative abundances of known PAOs and GAOs as indicated by 16S rRNA gene amplicon analysis with different primer sets annotated using the MiDAS databases, respectively.

To test the impact of primer selection on the community structure analyses, two primer sets, i.e. 27F’-534R (V1-V3) and 515F-926R (V4-V5), were used to analyze 26 activated sludge samples (1 full-scale sludge, 25 lab-scale reactor sludge) (Fig. 7 and Fig. S3, see online supplementary material for a colour version of this figure). The sequencing results were further annotated by using SILVA and MiDAS as reference databases, respectively, to understand the impact of annotation databases. For the same set of samples, 8774 and 11 727 ASVs were recovered with 27F’-534R and 515F-926R, respectively. Annotations with SILVA and MiDAS further resulted in different resolutions in different taxonomic units (Fig. S3, see online supplementary material for a colour version of this figure). Overall, the MiDAS database conferred higher resolutions than the SILVA database. On average, 125 (27F’-534R) and 258 (515F-926R), and 109 (27F’-534R) and 159 (515F-926R) genera were successfully annotated with the MiDAS and the SILVA databases, respectively. MiDAS annotation also resulted in increased assignments down to the species level (190 and 369 species for 27F’-534R and 515F-926R, respectively) than SILVA annotation (22 and 31 species correspondingly). Sequencing with 515F-926R resulted in a significantly high average relative abundance of *Tetrasphaera* (0.71%, with both SILVA and MiDAS) than with 27F’-534R (0.30% with SILVA and 0.41% with MiDAS). With SILVA, 27F’-534R resulted in higher average relative abundances of *Ca*. Accumulibacter, *Ca*. Competibacter, *Ca*. Contendobacter, *Defluviicoccus*, and *Dechloromonas* (at 0.33%, 11.8%, 0.35%, 0.67%, and 0.63%, respectively) than 515F-926R (0.07%, 10.02%, 0.02%, 0.21%, and 0.07%, respectively). Similar results were observed with the MiDAS database, where the obtained average relative abundances of *Ca*. Accumulibacter, *Ca*. Competibacter, *Ca*. Contendobacter, *Defluviicoccus*, and *Dechloromonas* were 1.82%, 19.23%, 0.28%, 0.98%, and 0.28%, respectively, for 27F’-534R, significantly higher than the values obtained with 515F-926R (0.07%, 9.63%, 0.02%, 0.21%, and 0.06%, respectively). These results agreed with the primer coverage results, where 515F-926R showed higher coverage of *Tetrasphaera* (92.1%) and lower coverage of *Ca*. Contendobacter (78.3%) than 27F'-534R (80.5% and 100% in the SILVA database, respectively). Whereas, the *in-silico* analysis showed that the coverages of 27F’-534R for *Ca*. Accumulibacter, *Ca*. Competibacter, *Defluviicoccus*, and *Dechloromonas* were lower than 515F-926R, which is out of keeping with the experimental results. For specific samples, the occurrence of specific strains/sequences is also a key factor determining the outcome of different primer sets. As a whole, these results suggest the analyses of the same samples using different primers produced distinct results. V1-V3 region resulted in higher resolution of the EBPR community in these activated sludge samples. In addition, by comparing the classification results obtained via annotation with two databases (Fig. 7), MiDAS was more powerful for *Ca*. Accumulibacter annotation and classification, a great deal of sequences, which were identified as unclassified sequences with SILVA, were successfully annotated as *Ca*. Accumulibacter with MiDAS. For the 16S rRNA gene amplicon sequencing results obtained with 515F-926R, 38 less *Ca*. Accumulibacter ASVs were annotated with SILVA than with MiDAS. For the results obtained with 27F’-534R, the ASVs assigned to *Ca*. Accumulibacter was 203 with MiDAS and 13 with SILVA, resulting in significantly underestimated relative abundances of *Ca*. Accumulibacter with SILVA (0%–1.25% versus 0%–6.06%).

### Rational interpretation of FISH and 16S rRNA gene amplicon sequencing results

Inconsistencies between FISH and 16S rRNA gene amplicon sequencing results were commonly observed. Two methods lead to distinct relative abundances for the same microorganism. For instance, Rubio-Rincón *et al*. [82] showed that *Tetrasphaera* was highly abundant as suggested by 16S rRNA gene amplicon sequencing. However, *Ca*. Accumulibacter remained as the predominant PAO in the FISH-biovolume-based quantitative analysis with PAOmix [82]. There could be several reasons for the observed inconsistency. The poor coverage of existing *Tetrasphaera* probes may be a key reason for lowered detection of *Tetrasphaera* with FISH. And *Ca*. Accumulibacter was typically considered to have larger cell biovolumes (2–3 μm), resulting in overestimated abundances in biovolume analysis [38], except the small biovolume observed for *Ca*. Accumulibacter iunctus, the FISH abundance of which was lower than expected based on amplicon sequencing [20]. Another reason might be non-target bacteria (e.g., *Propionivibrio*) which covered by PAOmix. Previous research suggested that the unsatisfied specificity of PAOmix resulted in incorrectly concluded *Ca*. Accumulibacter with coccoid- and rod-shaped morphologies between *ppk*1-defined genotypes when PAOmix was used in combine with type probes [20]. On the other hand, the relative abundance of *Ca*. Accumulibacter tend to be underestimated in 16S rRNA gene amplification sequencing due to a smaller 16S rRNA gene copy numbers (i.e. two) encoded in *Ca*. Accumulibacter genomes [10]. This coincides with a previous study of *Propionivibrio*-GAO, where, *Ca*. Accumulibacter and *Propionivibrio* were found to have a similar relative abundance in 16S rRNA gene amplicon sequencing and qFISH; whereas, FISH quantification showed 2-fold the relative abundance of *Ca*. Accumulibacter than amplicon sequencing [38]. Previous studies also suggested that 16S rRNA gene amplicon sequencing tends to overestimate the relative abundance of *Dechloromonas* by a factor of 10 over FISH-based quantification [33, 83]. The results obtained in this study suggested that the coverages of the probes targeting *Dechloromonas* were all extremely low (2.8%–22.1%), which might be a cause of low relative abundance values of *Dechloromonas* observed by FISH analysis.

In studies, FISH-based biovolume quantification of *Ca*. Accumulibacter showed promising agreement with the EBPR activities from different WWTPs. For instance, samples with high EBPR activity (anoxic/aerobic P-uptake rate) were shown to have high abundance of *Ca*. Accumulibacter as indicated by FISH in a study of Carlos et al. [84]. Similarly, high abundance of *Tetrasphaera* and *Ca*. Accumulibacter observed in samples via FISH quantification were often accompanied with high P release values [73, 85]. These results suggested that as long as the predominant species/strains in a specific community were well-capture by the FISH probes (and with the occurrence of limited numbers of out-group target bacteria), FISH is a powerful tool for *in-situ* occurrence and abundance analyses.

Above all, 16S rRNA gene copy numbers of the targeted bacteria and their accompany community members also potentially affect a correct reflection of the “true” abundance of targeted bacteria groups [86]. The abundances of bacteria with larger numbers of 16S rRNA genes tend to be overestimated in 16S rRNA gene amplicon sequencing and vice versa [87]. We performed a systematic analysis of available PAO and GAO genomes/MAGs. Typically, *Ca*. Accumulibacter and *Defluviicoccus* encoded two copies of 16S rRNA genes. *Tetrasphaera*, *Ca*. Phosphoribacter, *Ca*. Lutibacillus, *Microlunatus*, *Ca*. Contendobacter and *Ca*. Competibacter encoded one copy of 16S rRNA genes. *Dechloromonas* encoded 3–4 copies of 16S rRNA genes [88, 89]. All the numbers are lower than the average 16S rRNA gene copy numbers (i.e. 4.1) of bacteria in the activated sludge [2, 90], suggesting that the relative abundances of these commonly occurring PAOs and GAOs obtained via 16S rRNA gene amplicon sequencing were typically lower than their actual abundance in activated sludge samples.

## Supplementary Material

Supplementary_2-Scripts_ycae011

Supplementary_table_1_ycae011

Supplementary_table_5_ycae011

Supplementary_table_6_ycae011

Supplementary_table_7_ycae011

Supplementary_Text_1-4_Supplementary_Figure1-3_ycae011

Supplementary_table_2_ycae011

Supplementary_table_3_ycae011

Supplementary_table_4_ycae011

## Data Availability

All data generated or analyzed during this study are included in this published article and its supplementary information files.
