# Supplementary material for: Blind spots of universal primers and specific FISH probes for functional microbe and community characterization in EBPR systems
Source: ISME Commun. 2024 Jan 23;4(1):ycae011. doi: 10.1093/ismeco/ycae011 (PMC10958769; doi:10.1093/ismeco/ycae011)
Supplement: Supplementary_2-Scripts_ycae011 [file supplementary_2-scripts_ycae011.pdf]

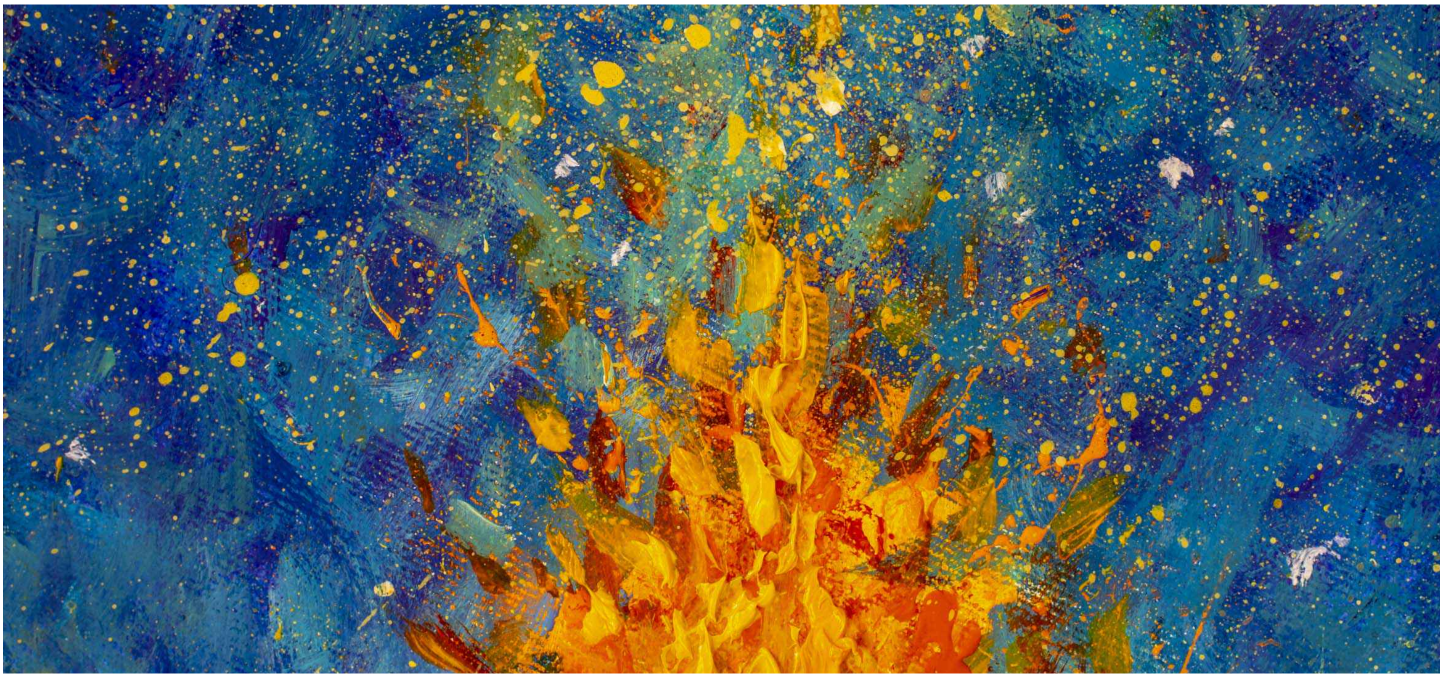

Supplementary 2 - Scripts

Online link of this script " Supplementary 2 - Scripts

Silva database alignment

Silva Database establishment

```
makeblastdb -dbtype nucl -in SILVA_138.1_SSURef_NR99_tax_silva.fasta  -out silva/silva -title silva -parse_seqids
```

Extract Silva sequence id and taxon name

```
conda activate seqkit
seqkit seq -n /Users/dengxuhan/db/silva/SILVA_138.1_SSURef_NR99_tax_silva.fasta | awk '{sub(/\ /,"\t");print}' > id_taxon.txt
```

Compare probe to silva database

```
#!/bin/bash
```

```
while getopts "i:o:d:h" opt; do
    case "$opt" in
        i) input_dir="$OPTARG" ;;
        o) output_dir="$OPTARG" ;;
        d) database="$OPTARG" ;;
        h)
            echo "please use -i to specify your input directory"
            echo "please use -o to specify your output directory"
            echo "use -d to specify the database position"
            exit 0
            ;;
        *)
            echo "wrong input, please use -h to find help"
            exit 1
            ;;
    esac
done
echo "Input directory: $input_dir"
echo "Output directory: $output_dir"
echo "Database position: $database"
mkdir -p "$output_dir/filter" "$output_dir/midfile" "$output_dir/result"
for file in "$input_dir"/*; do
    blastn -db "$database" -task blastn-short -query "$file" -outfmt "6 qseqid sseqid pident length mismatch gapopen qstart qend sstart send evalue bitscore qlen"  > "$output_dir/midfile/mid$(basename "$file")"
    awk '{if(($13==$4)&&($4==$8)&&($5==0)) {print $0}}' "$output_dir/midfile/mid$(basename "$file")" > "$output_dir/filter/filter$(basename "$file")"
    awk -F "\t" 'NR==FNR{a[$1]=$0;next} $2 in a {print a[$2]}' /Users/dengxuhan/db/silva/id_taxon.txt "$output_dir/filter/filter$(basename "$file")" >"${output_dir}/result/result$(basename "$file")"
done
```

example

```
bash silva.sh -i probe-Tet -o output -d /Users/dengxuhan/db/silva/silva/silva
```

Midas database align

Midas 4.0 BLAST database establishment

```
makeblastdb -dbtype nucl -in midas.fa  -out midas/midas -title midas
```

Compare primer to Midas 4.0

```
#!/bin/bash
mkdir mid
mkdir filter_blast
mkdir final_result
mkdir uniq
blastn -db /Users/dengxuhan/db/midas4.0/midas/midas -task blastn-short -num_descriptions 100000 -num_alignments 10000000 -query primer.fasta -outfmt "6 qseqid sseqid pident length mismatch gapopen qstart qend sstart send evalue bitscore qlen" | tee ./mid/mid_blast_result.txt | awk '{if(($13==$4)&&($4==$8)&&($5==0)) {print $0}}' - | tee ./filter_blast/filtered_blast_result.txt | awk '{print $2}' - | awk 'NR==FNR{a[$1]=$0;next}NR>FNR{if($1 in a)print a[$1]}' /Users/dengxuhan/db/midas4.0/midasfind.txt - >final_result/final_blast_result.txt
sort -n ./final_result/final_blast_result.txt | uniq > uniq/sort_blast_result.txt

cd uniq
for i in *;
do
cd $i
grep -wf *_B.fasta *_F.fasta > ${i}_merge.fasta
cd ..
done

for i in *.fasta;
do
grep 'g__Ca_Accumulibacter' $i > g__Ca_Accumulibacter.txt
grep 'f__Competibacteraceae' $i > f__Competibacteraceae.txt
grep 'g__Ca_Competibacter' $i > g__Ca_Competibacter.txt
grep 'g__Ca_Contendobacter' $i > g__Ca_Contendobacter.txt
grep 'g__Dechloromonas' $i > g__Dechloromonas.txt
grep 'g__Defluviicoccus' $i > g__Defluviicoccus.txt
grep 'g__Microlunatus' $i > g__Microlunatus.txt
grep 'g__Micropruina' $i > g__Micropruina.txt
grep 'g__Tetrasphaera' $i > g__Tetrasphaera.txt
grep 'g__Propionivibrio' $i > g__Propionivibrio.txt
echo ################### >> count.txtcount
echo $i >> count.txtcount
echo 'match'>> count.txtcount
```

```
wc -l *.txt >> count.txtcount
echo 'total'>> count.txtcount
wc -l $i >> count.txtcount
echo ############################### >> count.txtcount
cat *.txt > ${i}_cat_all.txta
done
```

Compare probe to Midas 4.0

```
#!/bin/bash
while getopts "i:o:d:r:h" opt; do
    case "$opt" in
        i) input_dir="$OPTARG" ;;
        o) output_dir="$OPTARG" ;;
        d) database="$OPTARG" ;;
        r) reference="$OPTARG" ;;
        h)
            echo "please use -i to specify your input directory"
            echo "please use -o to specify your output directory"
            echo "use -d to specify the database position"
            echo "use -r to specify midas reference file, doload from midas ,each row consume with midasID taxonomy"
            exit 0
            ;;
        *)
            echo "wrong input, please use -h to find help"
            exit 1
            ;;
    esac
done
echo "Input directory: $input_dir"
echo "Output directory: $output_dir"
echo "Database position: $database"
mkdir -p "$output_dir/filter" "$output_dir/midfile" "$output_dir/result"
for file in "$input_dir"/*; do
    blastn -db "$database" -task blastn-short -query "$file" -outfmt "6 qseqid sseqid pident length mismatch gapopen qstart qend sstart send evalue bitscore qlen" | tee "$output_dir/midfile/mid$(basename "$file")" | awk '{if(($13==$4)&&($4==$8)&&($5==0)) {print $0}}' - | tee "$output_dir/filter/filter$(basename "$file")" | awk '{print $2}' - | awk 'NR==FNR{a[$1]=$0;next}NR>FNR{if($1 in a)print a[$1]}' ${reference} - >"${output_dir}/result/result$(basename "$file")"
done
```

Extract specific species,genus sequence from Midas databse

```
#!/bin/bash
while getopts "p:m:n:o:" opt; do
  case "$opt" in
    p) midas_path="$OPTARG";;
    m) midastxt="$OPTARG";;
    n) organism="$OPTARG";;
    o) output="$OPTARG";;
    *) echo "-p Midas databse file -m midas ID-taxononmy file -n the taxononomy you want to extract e.g: g__Microlunatus  -o output dir $opt"
       exit 1;;
  esac
done
grep "$organism" $midastxt > $output/seq_species.txt
cut -f 1 $output/seq_species.txt > $output/seq.txt
seqkit grep -f $output/seq.txt $midas_path  -o $output/seq.fa
```
