# Supplementary material for: Blind spots of universal primers and specific FISH probes for functional microbe and community characterization in EBPR systems
Source: ISME Commun. 2024 Jan 23;4(1):ycae011. doi: 10.1093/ismeco/ycae011 (PMC10958769; doi:10.1093/ismeco/ycae011)
Supplement: Supplementary_table_1_ycae011 [file supplementary_table_1_ycae011.docx]

| **Table S1. Coverage and sequence of commonly used primer sets for 16S rRNA amplicon sequencing** | | | | | | | | | |
| --- | --- | --- | --- | --- | --- | --- | --- | --- | --- |
| **SILVA coverage (%)** | | | | | | | | | |
|  | Ca. Accumulibacter | Ca. Competibacter | Ca.Contendobacter | Dechloromonas | Defluviicoccus | Microlunatus | Micropruina | Tetrasphaera | Propionivibrio |
| 27F‘-533R | 59.3 | 80.4 | 100 | 68.8 | 67.9 | 0 | 0 | 31.4 | 64 |
| 27F-533R | 68.6 | 87.3 | 100 | 88.2 | 82.1 | 0 | 16.7 | 35.3 | 84 |
| 27F’-534R | 59.3 | 80.4 | 100 | 69.9 | 50 | 0 | 0 | 31.4 | 64 |
| 27F-534R | 68.6 | 85.7 | 100 | 89.2 | 60.7 | 0 | 16.7 | 35.3 | 84 |
| 338F-806R | 93 | 90.5 | 78.3 | 90.1 | 93.3 | 1.3 | 0 | 96.1 | 88.2 |
| 341F-806R | 93 | 90.5 | 78.3 | 90.6 | 93.3 | 1.3 | 0 | 96.1 | 88.4 |
| 515F-806R | 91.9 | 89.5 | 78.3 | 87.3 | 90.7 | 0 | 0 | 94.1 | 85.5 |
| 520F-802R | 93 | 92.6 | 95.7 | 87.3 | 96 | 1.3 | 0 | 100 | 86.8 |
| 515F-907R | 89.5 | 89.5 | 78.3 | 87.3 | 89.3 | 0 | 8.3 | 94.1 | 97.4 |
| 515F-926R | 91.9 | 89.5 | 78.3 | 87.8 | 90.7 | 0 | 8.3 | 94.1 | 97.4 |
| 799F-1193R | 87.2 | 89.5 | 87 | 86.2 | 88 | 1.3 | 0 | 74.5 | 90.8 |
| **MiDAS coverage (%)** | | | | | | | | | |
|  | Ca. Accumulibacter | Ca. Competibacter | Ca.Contendobacter | Dechloromonas | Defluviicoccus | Microlunatus | Micropruina | Tetrasphaera | Propionivibrio |
| 338F-806R | 100 | 100 | 100 | 99.7 | 100 | 0 | 0 | 100 | 100 |
| 341F-806R | 100 | 100 | 100 | 99.7 | 100 | 0 | 0 | 100 | 100 |
| 515F-806R | 100 | 100 | 5.4 | 100 | 100 | 0 | 0 | 100 | 100 |
| 520F-802R | 100 | 99.8 | 100 | 100 | 100 | 0 | 0 | 100 | 100 |
| 515F-907R | 100 | 100 | 100 | 100 | 100 | 0 | 0 | 100 | 100 |
| 515F-926R | 100 | 100 | 100 | 100 | 100 | 0 | 0 | 100 | 100 |
| 799F-1193R | 100 | 100 | 100 | 100 | 100 | 0 | 0 | 93 | 100 |
| **Sequence** | | | | | | | | | |
| 16S V1-3 | 27F’ | AGAGTTTGATCCTGGCTCAG | | | 533R | TTACCGCGGCTGCTGGCAC | | | [1][2] |
| 16S V1-3 | 27F | AGAGTTTGATCMTGGCTCAG | | | 533R | TTACCGCGGCTGCTGGCAC | | |  |
| 16S V1-3 | 27F’ | AGAGTTTGATCCTGGCTCAG | | | 534R | ATTACCGCGGCTGCTGGC | | |  |
| 16S V1-3 | 27F | AGAGTTTGATCMTGGCTCAG | | | 534R | ATTACCGCGGCTGCTGGC | | |  |
| 16S V3-4 | 338F | ACTCCTACGGGAGGCAGCA | | | 806R | GGACTACHVGGGTWTCTAAT | | | [3] |
| 16S V3-4 | 341F | CCTAYGGGRBGCASCAG | | | 806R | GGACTACNNGGGTATCTAAT | | | [4] |
| 16S V4 | 515F | GTGYCAGCMGCCGCGGTAA | | | 806R | GGACTACNVGGGTWTCTAAT | | | [5] |
| 16S V4 | 520F | AYTGGGYDTAAAGNG | | | 802R | TACNVGGGTATCTAATCC | | | [6] |
| 16S V4-5 | 515F | GTGCCAGCMGCCGCGGTAA | | | 907R | CCGTCAATTCMTTTRAGTTT | | | [7] |
| 16S V4-5 | 515F | GTGYCAGCMGCCGCGGTAA | | | 926R | CCGYCAATTYMTTTRAGTTT | | | [5] |
| V5-7 | 799F | AACMGGATTAGATACCCKG | | | 1193R | ACGTCATCCCCACCTTCC | | | [8] |
|  |  |  |  |  |  |  |  |  |  |
| 1. Albertsen M, Karst SM, Ziegler AS, Kirkegaard RH, Nielsen PH. Back to basics – the influence of DNA extraction and primer choice on phylogenetic analysis of activated sludge communities. *PLoS ONE* 2015; **10**: e0132783. | | | | | | | | | |
| 2. Jiang H, Dong H, Zhang G, Yu B, Chapman LR, Fields MW. Microbial Diversity in Water and Sediment of Lake Chaka, an Athalassohaline Lake in Northwestern China. *Appl Environ Microbiol* 2006; **72**: 3832–3845. | | | | | | | | | |
| 3.Dennis KL, Wang Y, Blatner NR, Wang S, Saadalla A, Trudeau E, et al. Adenomatous polyps are driven by microbe-instigated focal inflammation and are controlled by IL-10-producing T cells. *Cancer Res* 2013; **73**: 5905–5913. | | | | | | | | | |
| 4.Behrendt L, Larkum AWD, Trampe E, Norman A, Sørensen SJ, Kühl M. Microbial diversity of biofilm communities in microniches associated with the didemnid ascidian Lissoclinum patella. *The ISME journal* 2012; **6**: 1222–1237. | | | | | | | | | |
| 5. Walters W, Hyde ER, Berg-Lyons D, Ackermann G, Humphrey G, Parada A, et al. Improved Bacterial 16S rRNA Gene (V4 and V4-5) and Fungal Internal Transcribed Spacer Marker Gene Primers for Microbial Community Surveys. *mSystems* 2016; **1**: e00009-15. | | | | | | | | | |
| 6. Claesson MJ, O’Sullivan O, Wang Q, Nikkilä J, Marchesi JR, Smidt H, et al. Comparative Analysis of Pyrosequencing and a Phylogenetic Microarray for Exploring Microbial Community Structures in the Human Distal Intestine. *PLOS ONE* 2009; **4**: e6669. | | | | | | | | | |
| 7. Turner S, Pryer KM, Miao VPW, Palmer JD. Investigating Deep Phylogenetic Relationships among Cyanobacteria and Plastids by Small Subunit rRNA Sequence Analysis1. *Journal of Eukaryotic Microbiology* 1999; **46**: 327–338. | | | | | | | | | |
| 8. Horton MW, Bodenhausen N, Beilsmith K, Meng D, Muegge BD, Subramanian S, et al. Genome-wide association study of Arabidopsis thaliana leaf microbial community. Nat Commun 2014; 5: 5320. | | | | | | | | | |
