# Supplementary material for: Blind spots of universal primers and specific FISH probes for functional microbe and community characterization in EBPR systems
Source: ISME Commun. 2024 Jan 23;4(1):ycae011. doi: 10.1093/ismeco/ycae011 (PMC10958769; doi:10.1093/ismeco/ycae011)
Supplement: Supplementary_Text_1-4_Supplementary_Figure1-3_ycae011 [file supplementary_text_1-4_supplementary_figure1-3_ycae011.docx]

# *Supplementary Information*

# Blind spots of universal primers and specific FISH probes for functional microbe and community characterization in EBPR systems

Jing Yuan^1^ ([yuanjingyee@163.com](mailto:yuanjingyee@163.com)), Xuhan Deng^1^ ([dxh0222@gmail.com](mailto:dxh0222@gmail.com)), Xiaojing Xie^1^ (1030472309@qq.com), Liping Chen^1^ ([16121228@bjtu.edu.cn](mailto:16121228@bjtu.edu.cn)), Chaohai Wei^1,2,3^ ([cechwei@scut.edu.cn](mailto:cechwei@scut.edu.cn)), Chunhua Feng^1,2,3^ ([chfeng@scut.edu.cn](mailto:chfeng@scut.edu.cn)), Guanglei Qiu^1,2,3,^* ( [qiugl@scut.edu.cn](mailto:qiugl@scut.edu.cn))

^1^ School of Environment and Energy, South China University of Technology, 382 Waihuandong Road, University Town, Guangzhou, Guangdong, 510006, China

^2^ Guangdong Provincial Key Laboratory of Solid Wastes Pollution Control and Recycling, 382 Waihuandong Road, University Town, Guangzhou, Guangdong, 510006, China

^3^ Key Laboratory of Pollution Control and Ecological Restoration in Industrial Clusters, Ministry of Education, 382 Waihuandong Road, University Town, Guangzhou, Guangdong 510006, China

*Corresponding Author: Guanglei Qiu, School of Environment and Energy, South China University of Technology, 382 Waihuandong Road, University Town, Guangzhou, Guangdong, 510006, China

Competing interests

The authors declare no interests.

This supporting information includes 4 texts, 7 tables, and 3 figures.

# Supplementary Texts

# Text S1:

Detailed information about the scripts used in the article can be found in the PDF document Supplementary 2 – Scripts.

# Text S2. 16S rRNA gene amplicon sequencing

The bacterial 16S rRNA gene of 26 activated sludge samples were amplified with primer sets: 27F’ (5’-AGAGTTTGATCCTGGCTCAG-3’)-534R (5’-TTACCGCGGCTGCTGGCAC-3’) [1] and 515F (5’-GTGYCAGCMGCCGCGGTAA-3’)-926R (5’-CCGYCAATTYMTTTRAGTTT-3’) [2], respectively, using a thermocycler (GeneAmp 9700, ABI, US). Polymerase chain reaction (PCR) were performed in triplicates in a 25-μL system containing 5 μL of 5×reaction Buffer, 5 μL of 5×GC buffer, 2 μL of 2.5 mM dNTPs, 1 μL of each primer (10 μM), 8.75 μL of ddH_2_O, 0.25 μL of Q5 DNA polymerase and 2 μL of DNA template with the following program: 2 min of denaturation at 98^o^C, 30 S of annealing at 55^o^C, and 30 S of elongation at 72^o^C, and a final extension at 72^o^C for 5 min. The resultant PCR products were gel extracted (2% agarose) and purified using the AxyPrep DNA Gel Extraction Kit (Axygen Biosciences, USA). Purified and indexed amplicons were pooled and paired-end sequenced (2×300) on an Illumina MiSeq platform (Illumina, US) according to the standard protocols at Majorbio Bio-Pharm Technology Co. Ltd. (Shanghai, China). The generated data was filtered, trimmed and denoised, before the paired end sequences were merged using the DADA2 pipeline (Version 1.12) [3] to resolve the data to the level of amplicon sequence variants (ASVs) [4]. Taxonomy was assigned using SSU r138.1 and MiDAS 4 [5], respectively, for comparison.

# Text S3. Coverage and specificity of complementary probes for *Ca*. Accumulibacter

In addition to PAOmix [6], probes targeting different clades of *Ca*. Accumulibacter were designed (Main text Table 1). Acc444, HAcc466 and HAcc426 were used to target clade ІA [7]. Acc184, CAcc184 and Acc623 were used to target clade ⅡC [7, 8]. Acc119, HAcc99, HAcc139, and CAcc119 were used to target clade ⅡA [7]. The coverage of these clade-specific probes on *Ca.* Accumulibacter related sequences were shown in Fig. S3, among which only Acc184 and Acc623 showed 100% specificity. Acc184 covered a *Ca.* Accumulibacter sequence (JQ726366) which was not covered by PAOmix. Most recently, a set of species-level FISH probe (Main text Table 1) was designed to resolve *Ca*. Accumulibacter species with different morphologies, including Acc469, Acc471, Acc1011, Acc635, Acc470, Acc471_2, Acc213, Acc442, Acc441 [9]. Their coverage values in the SILVA database were 1.2%, 31.4%, 5.8%, 3.5%, 17.4%, 8.1%, 0.0%, 0.0%, and 0.0%, respectively. Acc469 showed 100% specificity. Acc471_2 covered 1 *Ca*. Accumulibacter (JQ726366) which were not covered by PAOmix. In addition, Acc471 covered 1 *Thauera*. Acc1011 covered 1 *Verticiella*, 1 *Parapusillimonas*, 1 GKS98 freshwater group, 2 *Sulfuritalea*, 1 *Zoogloea*, 1 *Arenimonas*, and 1 *Dechloromonas* (i.e., JN391838, which was not covered by existing *Dechloromonas* probes). Acc635 covered 3 *Dechlorosoma*. Acc470 covered 1 *Rhodocyclus*. Acc471_2 covered 1 *Dechlorosoma*. Acc213, Acc442, Acc441, and Acc469 covered no non-*Ca.* Accumulibacter sequences. In the MiDAS database, all these probes showed 100% specificity with coverage values of 0.9% (Acc469), 18.3% (Acc471), 1.6% (Acc1011), 3.2% (Acc635), 29.8% (Acc470), 9.6% (Acc471_2), 0.0% (Acc213), 0.0% (Acc442), and 0.0% (Acc441), respectively.

# Text S4. 16S rRNA gene sequences which were not covered by common probes in the SILVA database

There are 3 *Ca.* Accumulibacter sequences which were not covered by exciting probes. None of them were primarily retrieved from enhanced biological phosphorus removal (EBPR) systems (Table S2).

Sixteen *Tetrasphaera* reference sequences were not covered by any existing probes (Table S2). 6 of them were recovered from activated sludge samples. KJ808303 were obtained in an A/A/O system, with relative abundances up to 2.4% [10]. AF125092 and AF125091 were recovery from liquid medium [11]. GU552251 was obtained in full-scale WWTPs, with a relative abundance of 1.25% [12]. Y14595 was obtained from activated sludge with a relative abundance of 1.0 % [13].

A majority of *Dechloromonas* (63.7%, 116 out of 182) were not covered by existing probes (DCMAG455, DEMFE455 [14], Bet135 [15], Dech443 [16], and Dech453 [8]). A lot of them occurred in activated sludge (55 *Dechloromonas* sequences). For instance, AB166783 was recovered from activated sludge. KJ808078 was retrieved from an A/A/O system [10].

Five *Ca*. Competibacter related reference sequences (JQ211645, FPLM01006675, KF697440, FPLP01005528, and KP717500, Table S4) were not covered by GAO431, GAO989 [17] or CPB654 [18]. KP717500 [19] and KF697440 [20] were obtained from activated sludge samples. FPLM01006675 and FPLP01005528 were documented in 2016 with their recovered sources unknown.

70.7% *Defluviicoccus* (53 reference sequences) were not covered by existing probes (DEF988 [21], DF181A, DF181B [22], DF198, DF1013, DF1004 [23], TFO_DF862, TFO_DF618, TFO_DF218 [16], or DF1020)[21]. DQ413127 was recovered from an anaerobic/aerobic sequential batch reactor (SBR). AF179678 [24] and FJ356059 [25] were Cluster I *Defluviicoccus* occurred in a full-scale and a lab-scale EBPR systems, respectively. AY351635 and AY351639 were also Cluster I members recovered via cloning from an acetate-fed membrane bioreactor [26]. FJ623337, FJ623367, and FJ623386 occurred in an SBR with the alkaline fermentation liquid of waste activated sludge as an carbon source [27]. KF697555 and KF697476 were recovered in the study where *Ca.* Competibacter KF697440 (which was not covered by existing *Ca.* Competibacter FISH probes) was recovered [20].

**Supplementary Tables**

**Table S1.** Coverage and sequence of commonly used primer sets for 16S rRNA amplicon sequencing

**Table S2.** *Ca.* Accumulibacter 16S rRNA gene sequences in the SILVA database which were not covered by any existing FISH probes

**Table S3.** *Tetrasphaera* 16S rRNA gene sequences in the SILVA database which were not covered by any existing FISH probes

**Table S4.** *Dechloromonas* 16S rRNA gene sequences in the SILVA database which were not covered by any existing FISH probes

**Table S5.** *Ca.* Competibacter and *Ca.* Contendobacter 16S rRNA gene sequences in the SILVA database which were not covered by any existing FISH probes

**Table S6.** *Defluviicoccus* 16S rRNA gene sequences in the SILVA database which were not covered by any existing FISH probes

**Table S7.** *Ca.* Accumulibacter, *Tetrasphaera*, *Dechloromonas*, *Ca.* Competibacter, and *Defluviicoccus* 16S rRNA genes in the MiDAS database which were not covered by any existing FISH probes

# Supplementary Figures


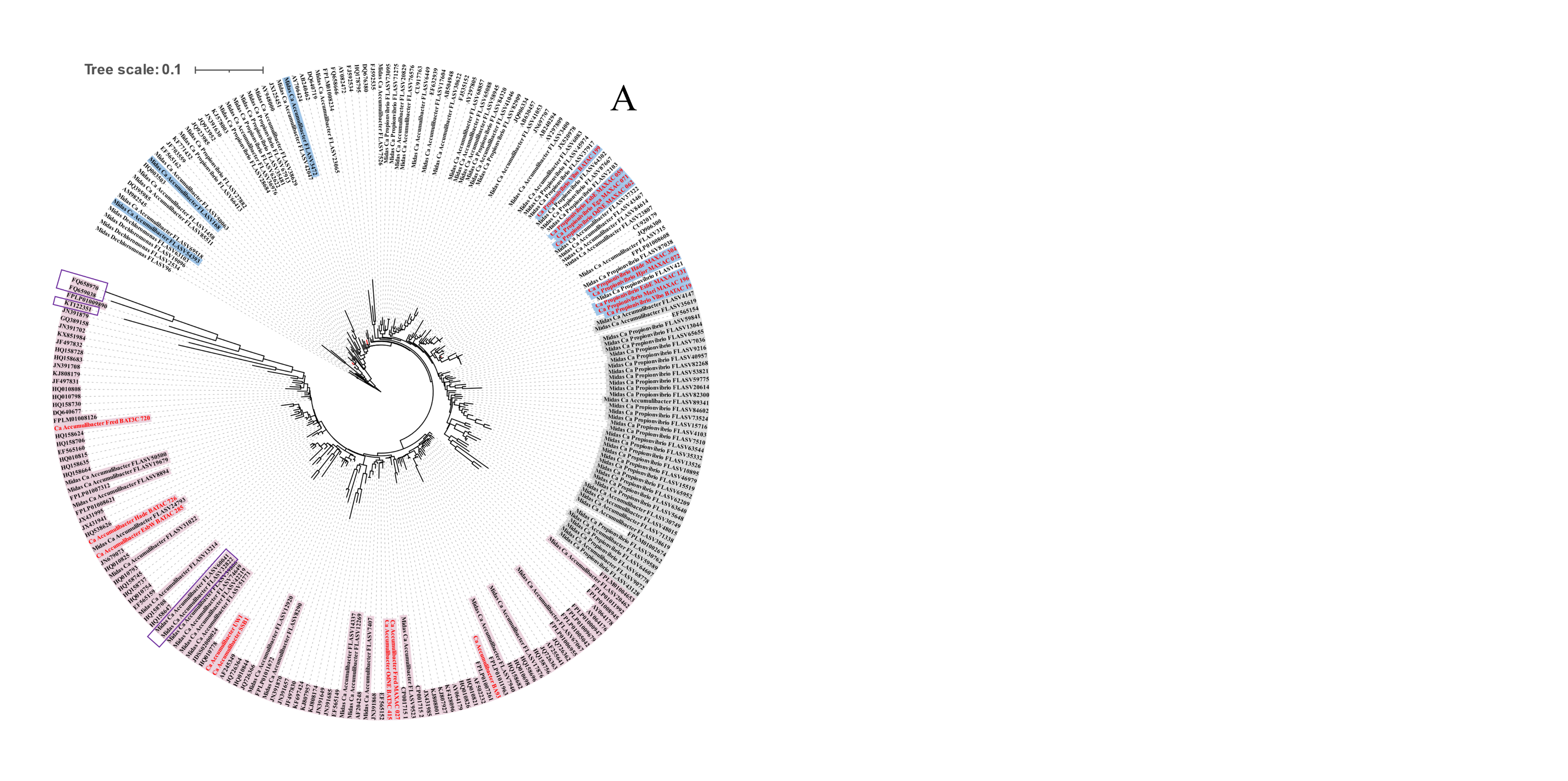


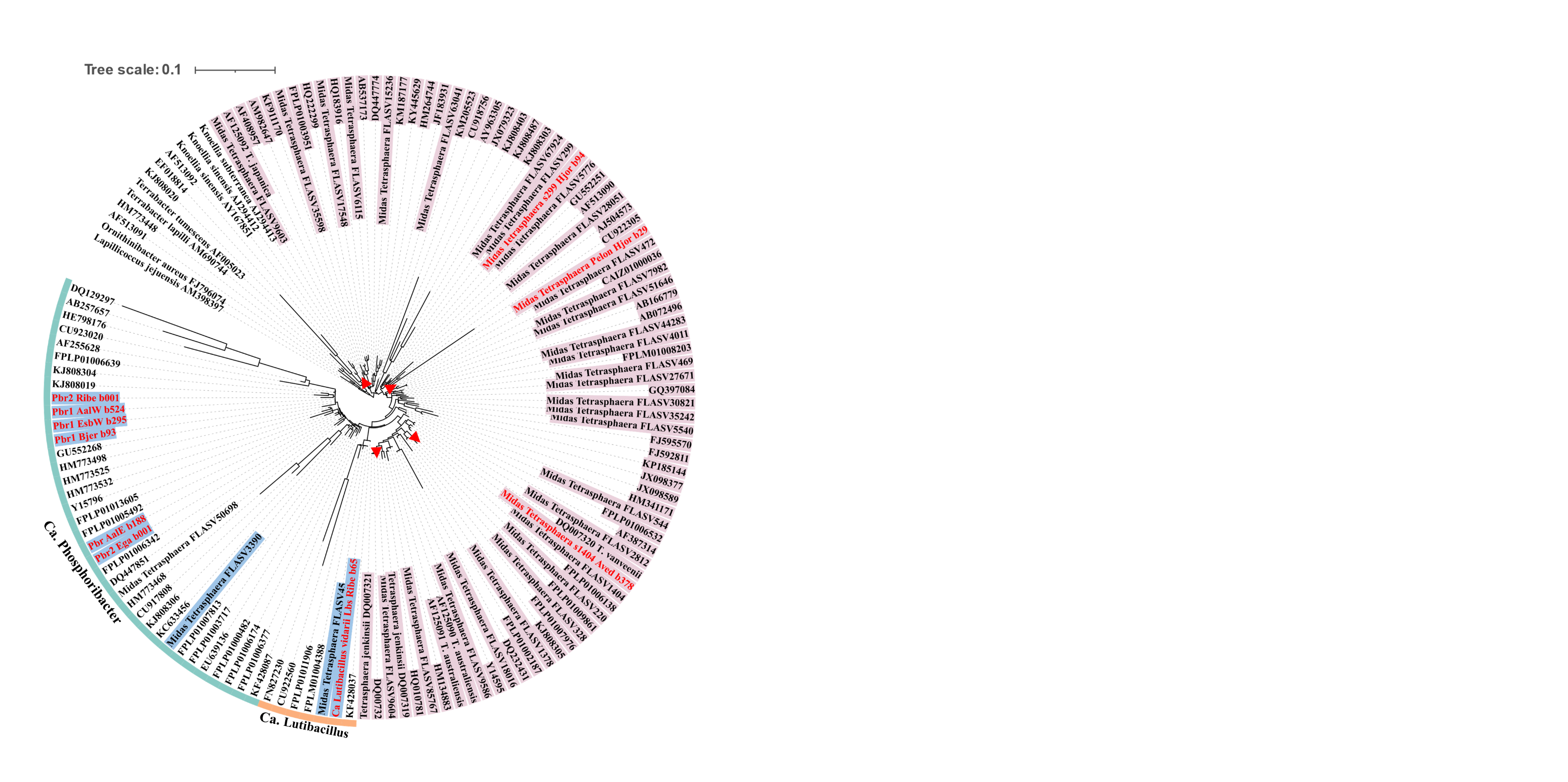


**Figure S1**. Phylogenetic analyses of (**A**) *Ca*. Accumulibacter- and *Propionvibrio*-related sequences, and (**B**) *Tertasphaera-*, *Ca.* Phosphoribacter- and *Ca.* Lutibacillus-related sequences. Leaves color-coded in red represent 16S rRNA gene sequences retrieved from metagenome-assembled genomes. Leaves in black are entries from the MiDAS and the SILVA databases. Leaves in blue represent identified non-*Ca*. Accumulibacter (or non-*Tertasphaera*) sequences in literatures [9, 28]. Clades in pink represent *Ca*. Accumulibacter (or *Tetrasphaera*) sequence. Clades in grey in panel A represent confirmed non-*Ca*. Accumulibacter sequences. Triangles in panel B represent identified sequences in literatures [9, 28]. Purple boxes in panel A represent sequences which are not covered by existing probes.

**Figure S2.** Comparison of the coverage of 27F or 27F’.

**Figure S3.** Number of taxonomic units at each level annotated in different databases.

**References**

1. Albertsen M, Karst SM, Ziegler AS, Kirkegaard RH, Nielsen PH. Back to basics – the influence of DNA extraction and primer choice on phylogenetic analysis of activated sludge communities. *PLoS ONE* 2015; **10**: e0132783.

2. Walters W, Hyde ER, Berg-Lyons D, Ackermann G, Humphrey G, Parada A, et al. Improved Bacterial 16S rRNA Gene (V4 and V4-5) and Fungal Internal Transcribed Spacer Marker Gene Primers for Microbial Community Surveys. *Msystems* 2016; **1**: e00009-15.

3. Callahan BJ, Mcmurdie PJ, Rosen MJ, Han AW, Holmes SP. DADA2: High resolution sample inference from amplicon data. *Cold Spring Harbor Laboratory* 2015.

4. Callahan BJ, McMurdie PJ, Holmes SP. Exact sequence variants should replace operational taxonomic units in marker-gene data analysis. *ISME J* 2017; **11**: 2639–2643.

5. Dueholm MKD, Nierychlo M, Andersen KS, Rudkjøbing V, Knutsson S, Albertsen M, et al. MiDAS 4: a global catalogue of full-length 16S rRNA gene sequences and taxonomy for studies of bacterial communities in wastewater treatment plants. *Nat Commun* 2022; **13**: 1908.

6. Crocetti GR, Hugenholtz P, Bond PL, Schuler A, Keller J, Jenkins D, et al. Identification of polyphosphate-Accumulating organisms and design of 16S rRNA-directed probes for their detection and quantitation. *Appl Environ Microbiol* 2000; **66**: 1175–1182.

7. Kim JM, Lee HJ, Kim SY, Song JJ, Park W, Jeon CO. Analysis of the fine-scale population structure of ‘Candidatus Accumulibacter phosphatis’ in enhanced biological phosphorus removal sludge, using fluorescence in situ hybridization and flow cytometric sorting. *Appl Environ Microbiol* 2010; **76**: 3825–3835.

8. Kim JM, Lee HJ, Lee DS, Jeon CO. Characterization of the Denitrification-Associated Phosphorus Uptake Properties of \"Candidatus Accumulibacter phosphatis\" Clades in Sludge Subjected to Enhanced Biological Phosphorus Removal. *Applied & Environmental Microbiology* 2013; **79**: 1969–1979.

9. Petriglieri F, Singleton CM, Kondrotaite Z, Dueholm MKD, McDaniel EA, McMahon KD, et al. Reevaluation of the phylogenetic diversity and global distribution of the genus “Candidatus Accumulibacter”. *mSystems* 2022; **7**: e00016-22.

10. Wang J, Li Q, Qi R, Tandoi V, Yang M. Sludge bulking impact on relevant bacterial populations in a full-scale municipal wastewater treatment plant. *Process Biochemistry* 2014; **49**: 2258–2265.

11. Maszenan AM, Patel BK, Schumann P, Burghardt J, Tokiwa Y, Stratton HM. Three isolates of novel polyphosphate-accumulating gram-positive cocci, obtained from activated sludge, belong to a new genus, Tetrasphaera gen. nov., and description of two new species, Tetrasphaera japonica sp. nov. and Tetrasphaera australiensis sp. nov. *Int J Syst Evol Micr* 2000; **50**: 593–603.

12. Hien, Thi, Thu, Nguyen, Vang, Quy, et al. High diversity and abundance of putative polyphosphate-accumulating Tetrasphaera-related bacteria in activated sludge systems. *FEMS Microbiol ecol* 2011; 256–267.

13. Blackall LL, Seviour EM, Bradford D, Rossetti S, Tandoi V, Seviour RJ. ‘Candidatus Nostocoida limicola’, a filamentous bacterium from activated sludge. *Int J Syst Evol Microbiol* 2000; **50**: 703–709.

14. Loy A, Schulz C, Lücker S, Schöpfer-Wendels A, Stoecker K, Baranyi C, et al. 16S rRNA gene-based oligonucleotide microarray for environmental monitoring of the betaproteobacterial order ‘Rhodocyclales’. *Appl Environ Microbiol* 2005; **71**: 1373–1386.

15. Kong Y, Xia Y, Nielsen JL, Nielsen PH. Structure and function of the microbial community in a full-scale enhanced biological phosphorus removal plant. *Microbiology* 2007; **153**: 4061–4073.

16. McIlroy SJ, Starnawska A, Starnawski P, Saunders AM, Nierychlo M, Nielsen PH, et al. Identification of active denitrifiers in full-scale nutrient removal wastewater treatment systems: Identifying active denitrifiers in activated sludge. *Environ Microbiol* 2016; **18**: 50–64.

17. Crocetti GR, Banﬁeld JF, Blackall LL. Glycogen-accumulating organisms in laboratory-scale and full-scale wastewater treatment processes. 2002; 12.

18. McIlroy SJ, Nittami T, Kanai E, Fukuda J, Saunders AM, Nielsen PH. Re-appraisal of the phylogeny and fluorescence in situ hybridization probes for the analysis of the Competibacteraceae in wastewater treatment systems. *Env Microbiol Rep* 2015; **7**: 166–174.

19. Chen Q, Ni J, Ma T, Liu T, Zheng M. Bioaugmentation treatment of municipal wastewater with heterotrophic-aerobic nitrogen removal bacteria in a pilot-scale SBR. *Bioresource Technology* 2015; **183**: 25–32.

20. Ittisupornrat S, Tobino T, Yamamoto K. A study of the relationship among sludge retention time, bacterial communities, and hydrolytic enzyme activities in inclined plate membrane bioreactors for the treatment of municipal wastewater. *Appl Microbiol Biotechnol* 2014; **98**: 9107–9118.

21. Meyer RL, Saunders AM, Blackall LL. Putative glycogen-accumulating organisms belonging to the Alphaproteobacteria identified through rRNA-based stable isotope probing. *Microbiology* 2006; **152**: 419–429.

22. Mcilroy SJ, Seviour RJ. Elucidating further phylogenetic diversity among the Defluviicoccus-related glycogen-accumulating organisms in activated sludge. *Env Microbiol Rep* 2009; **1**: 563–568.

23. Nittami T, McIlroy S, Seviour EM, Schroeder S, Seviour RJ. Candidatus Monilibacter spp., common bulking filaments in activated sludge, are members of cluster III Defluviicoccus. *Syst Appl Microbiol* 2009; **32**: 480–489.

24. Maszenan AM, Seviour RJ, Patel BKC, Janssen PH, Wanner J. Defluvicoccus vanus gen. nov., sp. nov., a novel Gram-negative coccus/coccobacillus in the ‘Alphaproteobacteria’ from activated sludge. *Int J Syst Evol Micr* 2005; **55**: 2105–2111.

25. McIlroy SJ, Tillett D, Petrovski S, Seviour RJ. Non-target sites with single nucleotide insertions or deletions are frequently found in 16S rRNA sequences and can lead to false positives in fluorescence *in situ* hybridization (FISH): Insertions/deletions can give false positives in FISH. *Environ Microbiol* 2011; **13**: 33–47.

26. Wong M-T, Tan FM, Ng WJ, Liu W-T. Identification and occurrence of tetrad-forming Alphaproteobacteria in anaerobic–aerobic activated sludge processes. *Microbiology* 2004; **150**: 3741–3748.

27. Jiang Y, Chen Y, Zheng X. Efficient Polyhydroxyalkanoates Production from a Waste-Activated Sludge Alkaline Fermentation Liquid by Activated Sludge Submitted to the Aerobic Feeding and Discharge Process. *Environ Sci Technol* 2009; **43**: 7734–7741.

28. Singleton CM, Petriglieri F, Wasmund K, Nierychlo M, Kondrotaite Z, Petersen JF, et al. The novel genus, ‘Candidatus Phosphoribacter’, previously identified as Tetrasphaera, is the dominant polyphosphate accumulating lineage in EBPR wastewater treatment plants worldwide. *ISME J* 2022; **16**: 1605–1616.
